# Antiplatelet Drug Ticagrelor Enhances Chemotherapeutic Efficacy by Targeting the Novel P2Y12-AKT Pathway in Pancreatic Cancer Cells

**DOI:** 10.3390/cancers12010250

**Published:** 2020-01-20

**Authors:** Omar Elaskalani, Alice Domenchini, Norbaini Binti Abdol Razak, Danielle E. Dye, Marco Falasca, Pat Metharom

**Affiliations:** 1 Platelet Research Laboratory, School of Pharmacy and Biomedical Sciences, Curtin Health and Innovation Research Institute, Faculty of Health Sciences, Curtin University, Bentley Campus, Kent Street, Bentley, Building 305, Perth, WA 6102, Australia; omar.elaskalani@postgrad.curtin.edu.au (O.E.); n.abdolrazak@postgrad.curtin.edu.au (N.B.A.R.); Danielle.dye@curtin.edu.au (D.E.D.); 2 Platelet Research Group, Perth Blood Institute, West Perth, WA 6005, Australia; 3 Metabolic Signalling Group, School of Pharmacy and Biomedical Sciences, Curtin Health Innovation Research Institute, Curtin University, Perth, WA 6102, Australia; alice.domenchini@curtin.edu.au (A.D.); marco.falasca@curtin.edu.au (M.F.); 4 Western Australian Centre for Thrombosis and Haemostasis, Health Futures Institute, Murdoch University, Perth, WA 6150, Australia

**Keywords:** antiplatelet drug, chemoresistance, P2Y12, pancreatic cancer, ADP

## Abstract

*Background:* Extensive research has reported that extracellular ADP in the tumour microenvironment can stimulate platelets through interaction with the platelet receptor P2Y12. In turn, activated platelets release biological factors supporting cancer progression. Experimental data suggest that the tumour microenvironment components, of which platelets are integral, can promote chemotherapy resistance in pancreatic ductal adenocarcinoma (PDAC). Thus, overcoming chemoresistance requires combining multiple inhibitors that simultaneously target intrinsic pathways in cancer cells and extrinsic factors related to the tumour microenvironment. We aimed to determine whether ticagrelor, an inhibitor of the ADP–P2Y12 axis and a well-known antiplatelet drug, could be a therapeutic option for PDAC. *Methods:* We investigated a functional P2Y12 receptor and its downstream signalling in a panel of PDAC cell lines and non-cancer pancreatic cells termed hTERT-HPNE. We tested the synergistic effect of ticagrelor, a P2Y12 inhibitor, in combination with chemotherapeutic drugs (gemcitabine, paclitaxel and cisplatin), in vitro and in vivo. *Results:* Knockdown studies revealed that P2Y12 contributed to epidermal growth factor receptor (EGFR) activation and the expression of SLUG and ZEB1, which are transcriptional factors implicated in metastasis and chemoresistance. Studies using genetic and pharmacological inhibitors showed that the P2Y12–EGFR crosstalk enhanced cancer cell proliferation. Inhibition of P2Y12 signalling significantly reduced EGF-dependent AKT activation and promoted the anticancer activity of anti-EGFR treatment. Importantly, ticagrelor significantly decreased the proliferative capacity of cancer but not normal pancreatic cells. In vitro, synergism was observed when ticagrelor was combined with several chemodrugs. In vivo, a combination of ticagrelor with gemcitabine significantly reduced tumour growth, whereas gemcitabine or ticagrelor alone had a minimal effect. *Conclusions:* These findings uncover a novel effect and mechanism of action of the antiplatelet drug ticagrelor in PDAC cells and suggest a multi-functional role for ADP-P2Y12 signalling in the tumour microenvironment.

## 1. Background

Pancreatic cancer has the lowest survival rates, where approximately 9% of patients survive five years after diagnosis [1]. Pancreatic ductal adenocarcinoma (PDAC) is the most common malignancy of the pancreas, accounting for >90% of pancreatic cancer cases [2]. Late diagnosis, the prevalence of metastasis and chemotherapy resistance account for the poor survival rates. Pancreatic cancer is also associated with thrombotic complications [3]. The elevated risk is attributed to the high metastatic rate and the ability of pancreatic cancer cells to activate platelets and the coagulation cascade [4]. The current standard of care for PDAC includes gemcitabine as a single agent or in combination with nab-paclitaxel. Another combination of chemotherapy, FOLFIRINOX (5-fluorouracil, oxaliplatin, irinotecan and leucovorin), has limited use in PDAC due to its profound adverse effects [5]. Anticoagulants have been examined as an adjuvant in combination with chemotherapy in PDAC, and despite a significant reduction in symptomatic venous thrombosis, there was no major increase in overall survival [6,7,8]. Recently, a clinical trial has been initiated to examine clopidogrel (antiplatelet, P2Y12 inhibitor) in combination with gemcitabine in PDAC patients (NCT02404363).

Several pathways in cancer cells can cause chemoresistance, including increased drug efflux, reduced drug cellular uptake, accelerated drug deactivation and activation of alternative oncogenic signalling pathways. Moreover, activation of the epithelial–mesenchymal transition (EMT), a cell developmental programme, in cancer cells further promotes chemotherapy resistance in PDAC [5]. We recently showed that platelet-derived factors, predominantly ADP/ATP, were responsible for an increased level of SLUG, an EMT transcriptional factor, which regulated the expression of cytidine deaminase (CDD) and equiliberative nucleoside transporter 1 (ENT1), and contributed to gemcitabine resistance [9].

The Gi-coupled P2Y12 receptor plays a crucial role in platelet function. P2Y12 downstream signalling in platelets is mediated through PI3-kinases, AKT, extracellular signal regulated kinases (ERK), Src kinases, small G protein Rap1 and G protein-gated inwardly rectifying potassium channels (GIRK) [10,11]. ADP is an important platelet agonist. It activates platelets through Gq-coupled P2Y1 and Gi-coupled P2Y12 receptors [12]. Solid tumours secrete more ADP (and ATP) compared to normal tissues, especially under hypoxia, which often occurs in solid tumours as they outgrow their blood supply [13]. ADP released by tumours activates platelets P2Y12 and P2Y1, which in turn releases growth factors to support tumour growth and metastasis [14]. P2Y12 is a target for several clinically available antithrombotic drugs [15]. Inhibition of platelet P2Y12 has been shown to reduce cancer growth and metastasis in ovarian, melanoma and lung cancer mice models [14,16]. However, the expression and signalling of P2Y12 in cancer cells are poorly investigated. The receptor is primarily expressed in platelets and brain tissue, with some reports showing P2Y12 in glioma, astrocytoma and breast cancer cell lines [17]. Here, we validated the expression of a functional P2Y12 in a panel of PDAC cells. Since several P2Y receptors have been shown to activate oncogenic EGFR signalling [18,19,20], we hypothesised that inhibition of P2Y12 may reduce epidermal growth factor receptor (EGFR) signalling and cancer growth. Our results show that ticagrelor, a clinically available P2Y12 inhibitor, exerted an anticancer effect and synergised with chemotherapeutic agents in vitro and in vivo.

## 2. Methods and Materials

### 2.1. Cell Lines

AsPC-1, BxPC-3, MiaPaCa-2, CFPAC-1, PANC1 and hTERT-HPNE cell lines were from ATCC^®^ (Manassas, VA, USA). The murine Kras-driven pancreatic cancer cell line MT4-2D was kindly provided by Professor David. Tuveson (Cold Spring Harbor Laboratory, Cold Spring Harbor, NY, USA). All cells tested negative for *Mycoplasma*. AsPC-1 and BxPC-3 cells were maintained in RPMI-1640 medium. MiaPaCa-2, PANC-1 and MT4-2D were maintained in DMEM medium. CFPAC-1 cells were maintained in IMDM medium. All culture media were supplemented with 10% fetal bovine serum (FBS, from Bovogen Biologicals, Melbourne, Australia), 2 mM glutamine, 1 mM sodium pyruvate and 1 mM non-essential amino acids. hTERT-HPNE cells were maintained in DMEM medium supplemented with 5% FBS, human epidermal growth factor (EGF 10 ng/mL, ThermoFischer, Waltham, MA, USA), puromycin (750 ng/mL, ThermoFischer, Waltham, MA, USA) and 5 mM D-glucose. Unless specified, all reagents were obtained from Gibco^®^ Life Technologies (Melbourne, Australia).

### 2.2. Reagents

Paclitaxel, ADP, Apyrase, Tween-80 and polyethylene glycol-300 (PEG-300) were purchased from Sigma-Aldrich, (St. Louis, MO, USA). Cisplatin and erlotinib were obtained from Selleckchem (Pittsburgh, PA, USA). Gemcitabine was obtained from Eli Lilly (Indianapolis, IN, USA). Ticagrelor was obtained from Sigma-Aldrich, Selleckchem and Pure Chemistry Scientific Inc. (Burlington, MA, USA). PSB-0739 and MRS 2179 were from Tocris Bioscience (Bristol, UK). Cultrex basement membrane Type-3 was from Trevigen, Inc. (Minneapolis, MN, USA). Matrigel was obtained from Corning Life Sciences (Corning, NY, USA)

### 2.3. Proliferation Assay

Cancer cells were seeded at 2000 cells per well in a 96-well plate. After 24 h, the media were replaced with fresh media (1% FBS) supplemented with different treatments for 72 h. In an experiment to examine the selectivity of ticagrelor on cancer versus normal cells, AsPC-1 cells were seeded in the same culture media used for hTERT-HPNE (except for puromycin), and both cell lines were treated with ticagrelor under the same experimental conditions. Cell viability was measured by detecting the metabolic activity of live cells using the tetrazolium dye MTT as previously described [21]. The half-maximal inhibitory concentration (IC_50_, µM) of ticagrelor was calculated using GraphPad Prism 8.0 Software (San Diego, CA, USA). For combination studies, a suboptimal dose of ticagrelor that is clinically relevant (2.5 µM) was chosen based on cell viability studies with ticagrelor on different cell lines. Synergism analysis was performed using CompuSyn Version 1.0 software (ComboSyn, Inc., Paramus, NJ, USA) based on Chou–Talalay’s combination index (CI) method [22].

### 2.4. Immunoblotting

The following specific antibodies against SLUG, ZEB1, p-AKT (Ser473), p-ERK1/2 (Thr202/Tyr204), EGFR, p-EGFR (Tyr1068) and α-actinin were obtained from Cell Signalling Technology^®^ (Danvers, MA, USA). Anti-P2Y12 antibodies were as follows; EPR18611 (Abcam, Cambridge, UK), 4H5L19 (ThermoFischer), and NBP2-61749 (Novus Biologicals, Littleton, CO, USA). Rabbit anti-CDD was obtained from Santa Cruz Biotechnology (Dallas, TX, USA).

In order to validate P2Y12 expression, pancreatic cells and washed human platelets were lysed in non-ionic detergent (1% n-dodecyl β-D-maltoside, 150 mM NaCl, 25 mM Tris·HCl, pH 7.5) supplemented with protease inhibitors (Cell Signaling Technology, Danvers, MA, USA). Cell lysates were kept at 4 °C for 2 h, then clarified using centrifugation. Twenty micrograms of protein from cell lysates, and ten micrograms from the platelet lysate were loaded per lane. Proteins were analysed using immunoblotting after an SDS-PAGE. It is important to mention that P2Y12 in the positive control (i.e., platelets) and test cell lines were subjected to the same experimental conditions to prepare lysates. P2Y12 can form homo, oligomers and heterodimers, and each form can be glycosylated and thus may appear at different molecular weights in immunoblots based on experimental conditions, cell types and detecting antibodies [23,24,25,26,27].

To investigate the P2Y12 signalling, cancer cells (1.5 × 10^5^ per well) were seeded in a 12-well plate. After 24 h, cells were serum-starved for 6 h. Different treatments were added and the plate was incubated for the specified time. Cells were lysed in a sample loading buffer (4% SDS, 20% glycerol, 10% 2-mercaptoethanol, 0.004% bromophenol blue and 0.125 M Tris HCl, pH 6.8) supplemented with protease/phosphatase Inhibitor Cocktail (Cell Signaling Technology^®^, Danvers, MA, USA). Lysates were then analysed using SDS–PAGE and immunoblotted for the relevant protein. Densitometry readings maybe found in supplemental data (Figure S6).

### 2.5. P2Y12 Knockdown

AsPC-1 cells were seeded at 5 × 10^4^ cells per well in a 12-well plate and maintained for 24 h. P2Y12 siRNA (Hs_P2RY12_4 FlexiTube Predesigned siRNA directed against human P2RY12, (NM_022788, NM_176876)) was obtained from Qiagen (Hilden, Germany). Silencer^®^ negative control (sequence: proprietary, catalogue #AM4635) was obtained from Ambion^®^ (ThermoFisher). P2Y12 and Silencer siRNA (25 nM) were prepared using a DharmaFECT1 transfection agent (1 µL per well) (Dharmacon, Lafayette CO, USA) in serum/antibiotic-free media. The transfection mixture was then added to the adherent cells in serum-free media. For BxPC-3, cells were seeded at 1.5 × 10^5^ cells per well in complete media in a 6-well plate. P2Y12 siRNA and Silencer (50 nM) were prepared using a DharmaFECT1 transfection agent (1 µL per well) in serum/antibiotic-free media. The transfection mixture was then added and the plate was incubated. After 24 h, the media were then replaced with complete media and incubated for 48 h. Cells were washed in PBS and then lysed with radioimmunoprecipitation assay buffer (RIPA) containing protease inhibitors).

### 2.6. ADP Secretion Assay

Cells were seeded at 7.5 × 10^5^ cells per well in a 6-well plate and grown overnight (12 h). After removing the media and washing cells, PBS (250 µL) was added, and cells were incubated for 15 min. PBS was then collected in ice-cold microcentrifuge tubes and spun at 300× *g* for 2 min at 4 °C. Supernatants were transferred to a black 96-well plate. ADP was included to ensure the selectivity of the assay. ADP was measured using an ADP assay kit (Abcam) according to the manufacturer’s instructions. Fluorescence was measured at Ex/Em 535/587 nm using a plate reader (EnSpire Multimode, PerkinElmer^®^, Waltham, MA, USA).

### 2.7. Apoptosis Assay

Cells were seeded at 3000 cells per well in a 96-well plate. After 24 h, ticagrelor was added (1, 5 and 10 µM) to the cells and incubated for 12 h. Apoptosis was evaluated using an Amplite fluorometric Caspase-3/7 Assay Kit (AAT Bioquest, Sunnyvale, CA, USA) according to the manufacturer’s instructions. The increase of Ex/Em = 350/450 nm was measured using the EnSpire Multimode plate reader. A NucView^®^ 488 Caspase-3 Assay Kit (Biotium, Fremont, CA, USA) was used to detect caspase-3 activity within live cells.

### 2.8. In Vivo Tumour Growth

Female NOD-SCID and C57BL6 mice aged 5–6 weeks were from the Animal Resources Centre (Murdoch, WA, Australia) and housed in specific pathogen-free conditions at the Life Science Research Facility, Curtin University. All experiments were performed according to the Australian Code of Practice as per the University Animal Ethics Committee (approval number ARE2018-34). A BxPC-3 xenograft model was established via the subcutaneous injection of 2.5 × 10^6^ cells in 100 µL RPMI/Cultrex basement membrane Type-3 (1:1) in the right flank of NOD-SCID mice. When tumours became palpable (50–100 mm^3^), the animals were randomly divided into four groups (vehicle control, ticagrelor, gemcitabine, ticagrelor plus gemcitabine). For the syngeneic model, MT4-2D cells (2.5 × 10^5^) in 100 µL RPMI/Matrigel (1:1) were injected in the right flank of female C57BL6 mice as previously described [28]. After two days, the mice were randomly divided into four groups (vehicle control, ticagrelor, gemcitabine, ticagrelor plus gemcitabine). Ticagrelor (50 mg/kg) was prepared in 4% DMSO, 30% PEG + 5% Tween 80 + ddH2O.

Gemcitabine (25 mg/kg) was prepared in 0.9% NaCl. Mice were given either ticagrelor or a vehicle via oral gavage (200 µL) twice a day every 12 h, five days a week (Monday–Friday) in addition to either gemcitabine or 0.9% NaCl via intraperitoneal injection (IP, 150 µL) once a week. The tumour diameters were monitored with a surgical calliper every three days. Tumour volumes were calculated using the formula = (width^2^ × length)/2.

### 2.9. Transforming Growth Factor Beta 1 (TGF-β1) ELISA

Mouse blood was collected via vena cava (under anesthetics) into ethylenediaminetetraacetic acid (EDTA, 5 mM final concentration). Plasma was prepared by centrifuging the sample for 15 min at 1500× *g* at 4 °C without brake, aliquoted and stored at −80 °C until further analysis. The levels of TGF-β1 were measured using a Mouse TGF-β1 ELISA Kit (Biosensis BEK-2095-1P). Samples were subjected to acid activation according to the manufacturer’s instructions. TGF-β1 concentrations in the samples were calculated from a standard curve generated at 450 nm using a plate reader.

### 2.10. Statistical Analysis

Data were analysed using GraphPad PRISM 8.0 software (GraphPad Software, San Diego, CA, USA). Results are expressed as the mean ± standard error (SEM). Student’s *t*-test, one-way ANOVA or two-way ANOVA were used to examine the significance of the mean as appropriate and as indicated in the figure legend. The *p*-values and statistical significance are reported as per the style of New England Journal of Medicine: <0.033 (*), <0.002 (**) and <0.001 (***).

### 2.11. Ethics Approval

The studies involving the use of animals were approved by the Curtin University Ethics committee (approval number ARE2018-34).

## 3. Results

### 3.1. P2Y12 Expression in PDAC Cells

**Figure 1:**
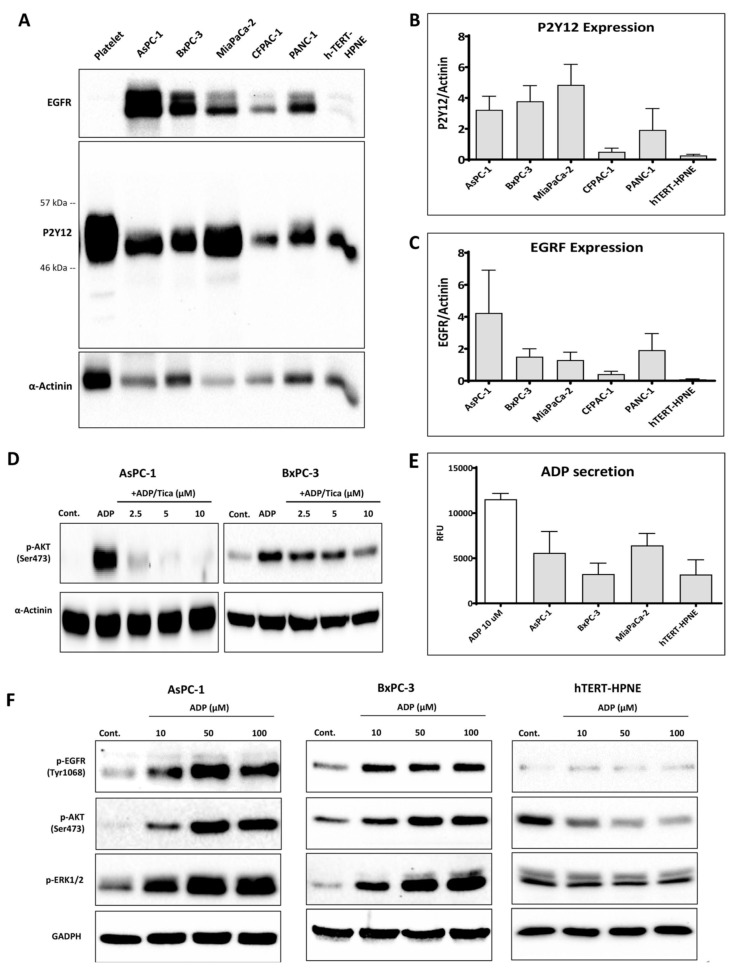
The P2Y12 receptor, activated by ADP, triggers AKT activation in PDAC cells. (**A**) Immunoblots show the expression of P2Y12 and EGFR in PDAC cells (AsPC-1, BxPC-3, MiaPaCa-2, CFPAC-1 and PANC-1) and the normal pancreatic duct cells h-TERT-HPNE. Platelets were used as a positive control for P2Y12. Cells were seeded at 3 × 10^5^ cells/well in a 6-well plate for 24 h, then washed, lysed and the proteins were collected and quantified. (**B**,**C**) Relative P2Y12 and EGFR expression in five PDAC cell lines and h-TERT-HPNE cells. The expression level was quantified and normalised to the loading control, α-actinin, with automated software Image Lab (version 5.1, BioRad, Hercules CA, USA) and represented as columns using GraphPad Prism 8 (GraphPad Software, Inc, CA, USA). (**D**) The P2Y12 inhibitor, ticagrelor, reduced ADP-induced AKT activation in AsPC-1 and BxPC-3. Briefly, cancer cells were seeded in a 12-well plate and after 24 h, cells were starved for 6 h, then treated with ticagrelor (5 µM) combined with ADP (100 µM) and the cells were further incubated for 30 min in serum-free media. The figure shows a representative blot from three independent experiments. (**E**) Extracellular ADP release from AsPC-1, BxPC-3, MiaPaCa-2 and h-TERT-HPNE. ADP was analysed in 250 µL of PBS previously incubated with cells for 15 min as described in the Methods and Materials section. The columns represent the mean of relative fluorescence units (RFU) from three independent experiments. (**F**) Western blot analysis of phospho-EGFR Y1068 (p-EGFR Y1068), phospho-AKT S473 (p-AKT S473) and phospho-ERK 1/2 (p-ERK1/2) expression in lysates derived from AsPC-1, BxPC-3 and h-TERT-HPNE treated with ADP (10, 50 and 100 µM) for 30 min. The figure shows a representative blot from three independent experiments.

P2Y12 expression in cancer and normal pancreatic ductal epithelial cells was detected at the same molecular weight as P2Y12 in platelets with an anti-P2Y12 monoclonal EPR18611 (Figure 1A). The results were confirmed using two additional different anti-P2Y12 antibodies (4H5L19 and NBP2-61749, Figure S1A). P2Y12 protein expression level was measured relative to α-actinin (Figure 1B). P2Y12 was overexpressed (>5-fold difference) in the PDAC cell lines AsPC-1, BxPC-3, MiaPaCa-2 and PANC-1 when compared to normal cells, hTERT-HPNE. The P2Y12 expression was the lowest in the well-differentiated cell line CFPAC-1 compared to those that were poorly differentiated (AsPC-1, MiaPaCa-2, PANC1) or the moderate-to-poorly differentiated BxPC-3 cell line. Similarly, EGFR was highly expressed in all PDAC cell lines compared to hTERT-HPNE (Figure 1C). A positive correlation between P2Y12 and EGFR expression in PDAC (R = 0.49, *p*-value = 2 × 10^−12^) was also demonstrated by the gene expression profiling interactive analysis (GEPIA) (Figure S4A) [29].

Since AKT is phosphorylated downstream of P2Y12 activation in platelets and glioma C6 cells [30,31], we investigated ADP-induced AKT signalling in PDAC cells. Our results showed that P2Y12 inhibitors, ticagrelor (Figure 1D) and PSB-0739 (Figure S1B), but not a P2Y1 antagonist (MRS 2179, Figure S1C) markedly reduced ADP-induced phosphorylated-AKT. Additionally, we measured the level of extracellular ADP secreted by PDAC cells and normal pancreatic cells, hTERT-HPNE; however, both tumour and non-tumour pancreatic cells produced comparable ADP secretion (Figure 1E). Since ADP, ATP and adenosine are known to induce transactivation of the mitogenic receptor EGFR [13], we hypothesised that ADP may induce prosurvival signals in tumour cell lines, but not in hTERT-HPNE, which expresses a relatively low level of P2Y12 and EGFR. To test this hypothesis, AsPC-1, BxPC-3 and hTERT-HPNE were treated with ADP. As a result, the activation of EGFR, AKT and ERK was significantly enhanced in PDAC cells, but not in hTERT-HPNE (Figure 1F).

### 3.2. P2Y12 Regulated Phospho-EGFR, PDAC Cell Proliferation and EMT Markers SLUG and ZEB1 

**Figure 2:**
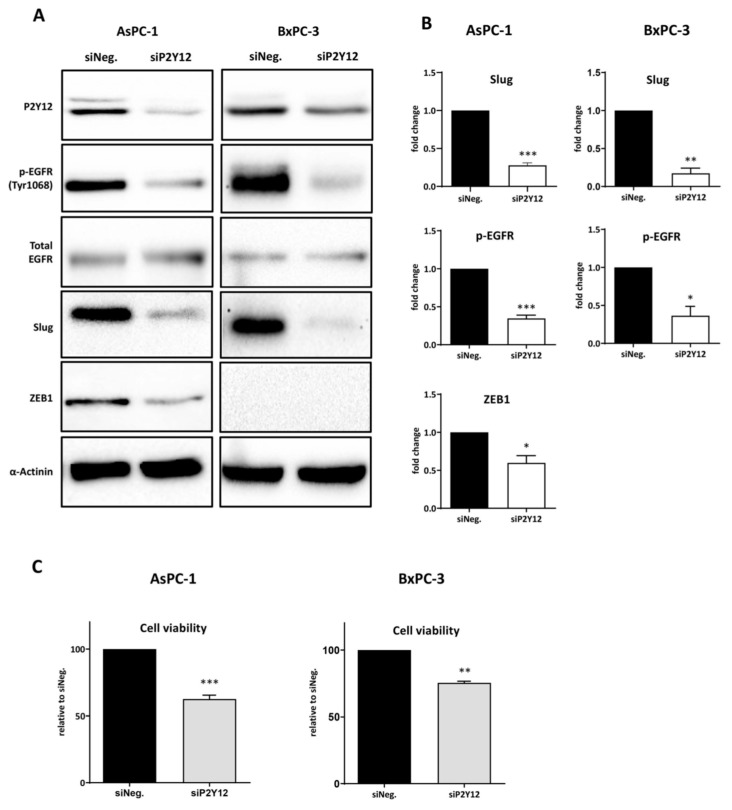
P2Y12 regulates EGFR activation, SLUG and ZEB1 expression and enhances PDAC cells viability. (**A**) Immunoblots showing the expression of P2Y12, p-EGFR Y1068, EGFR, SLUG and ZEB1 in AsPC-1 and BxPC-3 after P2Y12-specific siRNA treatment. Cancer cells were seeded and treated with P2Y12 siRNA or a negative control siRNA, as described in the Methods and Materials section. (**B**) The columns represent the fold change of protein levels (p-EGFR Y1068, SLUG and ZEB1) relative to the negative control siRNA-treated cells, normalised to 1 (*n* ≥ 3). Statistical analysis was performed using one sample *t*-test (GraphPad Prism 8) comparing the mean of siP2Y12 treatment with the normalised value 1. (**C**) Cell viability of AsPC-1 and BxPC-3 cells following knockdown of P2Y12 compared with the negative control siRNA-treated cells (*n* ≥ 3), calculated using one sample *t*-test (Graphpad Prism 8) and the normalised control group mean of 100%. Data are presented as mean ± SEM. *** *p* < 0.001, ** *p* < 0.002, * *p* < 0.033. siNeg: siRNA negative control.

Since we observed that ADP induced EGFR activation in PDAC cells, we wanted to assess whether P2Y12 is required for EGFR phosphorylation. We first investigated four different siRNA sequences (Figure S2A,B) and selected the best-performing sequence (Hs_P2RY12_4 FlexiTube) for subsequent studies. Immunoblot results showed that P2Y12-siRNA significantly reduced the level of p-EGFR in AsPC-1 and BxPC-3 cells grown in complete media (Figure 2A,B), and attenuated the proliferation of AsPC-1 and BxPC-3, compared to control siRNA transfected cells (Figure 2C). Additionally, P2Y12 knockdown significantly reduced the expression of EMT transcription regulators SLUG and ZEB1 in AsPC-1, and the SLUG level in BxPC-3 (Figure 2A,B).

### 3.3. Ticagrelor Reduced EGF-Induced AKT Activation in PDAC Cells

**Figure 3:**
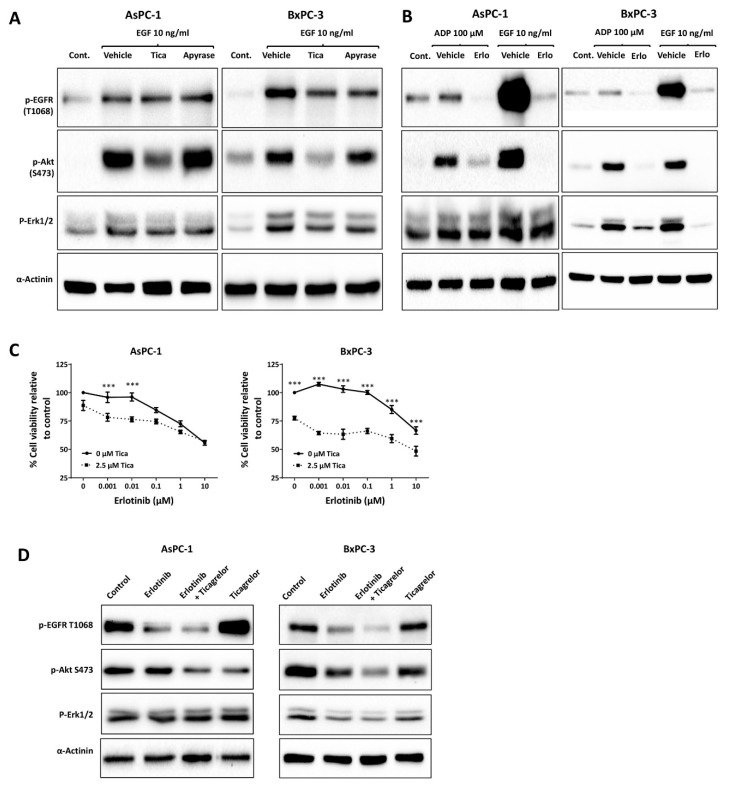
Ticagrelor attenuated EGF-stimulated AKT activation and potentiated the anticancer activity of erlotinib. (**A**) Immunoblots showing the expression of p-EGFR Y1068, p-AKT S473 and p-ERK1/2 in serum-starved AsPC-1 and BxPC-3 cells treated with EGF (10 ng/mL) combined with ticagrelor (5 µM) or apyrase (5 U/mL) for 1 h. (**B**) Immunoblots showing the expression of p-EGFR Y1068, p-AKT S473 and p-ERK1/2 in serum-starved AsPC-1 and BxPC-3 cells treated with ADP (100 µM) combined with erlotinib (5 µM) for 30 min. EGF +/− erlotinib was used as a control for EGFR activation. (**C**) Cell viability of AsPC-1 and BxPC-3 following treatment with erlotinib (0.001–10 µM) with and without ticagrelor (2.5 µM) for 72 h. Data are presented as mean ± SEM. Two-way ANOVA with post-hoc Bonferroni’s multiple comparison test (GraphPad PRISM 8.0) was used to examine the significance of the mean; *n* = 5, *** *p* < 0.001. Table 1A,B show the combination index (CI) values calculated using Chou–Talalay’s method and can be interpreted as follow: CI < 0.9, synergism, CI > 1.1, antagonism, CI = 0.9–1.1, additive. (**D**) Immunoblots show the expression of p-EGFR Y1068, p-AKT S473 and p-ERK1/2 in non-starved AsPC-1 and BxPC-3 cells treated with erlotinib (5 µM) combined with ticagrelor (2.5 µM) for 30 min. Immunoblots in (A), (B) and (D) are representative samples of at least three independent experiments.

P2Y12 is known to signal through AKT in platelets. As a result, P2Y12 inhibition reduces AKT activation in response to a variety of platelet agonists [30]. Therefore, we hypothesised that in PDAC cells, the inhibition of P2Y12 may reduce AKT activation in response to EGF. As shown in Figure 3A and Figure S1D, P2Y12 inhibitors ticagrelor (5 µM) and PSB-0739 (20 µM) reduced AKT and ERK activation in response to EGF (10 ng/mL, 30 min), while apyrase (5 U/mL) failed to show a significant effect. Ticagrelor and apyrase did not show a consistent inhibition of EGF-mediated EGFR phosphorylation. To further investigate P2Y12-EGFR crosstalk, we examined whether inhibition of EGFR may reduce ADP-mediated AKT activation. Erlotinib, an EGFR inhibitor, at 5 µM markedly reduced ADP-induced EGFR and AKT phosphorylation in AsPC-1 and BxPC-3 (Figure 3B). Since AKT is downstream of both EGFR and P2Y12, we tested whether ticagrelor can potentiate the anticancer activity of erlotinib. Figure 3C shows that the addition of ticagrelor increased the inhibitory effect of erlotinib. The synergism was evaluated using the CI method [22] (Table 1A,B). The CI values are classified as follows: 0.1–0.3 strong synergism, 0.3–0.7 synergism, 0.7–0.9 moderate to slight synergism, 0.9–1.1 nearly additive, 1.1–1.45 slight to moderate antagonism, 1.45–3.3 antagonism, and >3.3 strong to very strong antagonism [32]. At erlotinib concentrations of 0.001–1 µM, there was synergism, with CI values of less than 0.7 in AsPC-1 cells. In BxPC-3 cells, the CI values showed a slight synergism to nearly additive (CI 0.8–1.1) at 0.001–10 µM of erlotinib (Figure 3C). Immunoblot results showed that ticagrelor potentiated erlotinib-mediated AKT inhibition in AsPC-1 and BxPC-3 cells grown in 1% FBS (Figure 3D).

### 3.4. Ticagrelor Suppresses PDAC Cell Growth In Vitro

**Figure 4:**
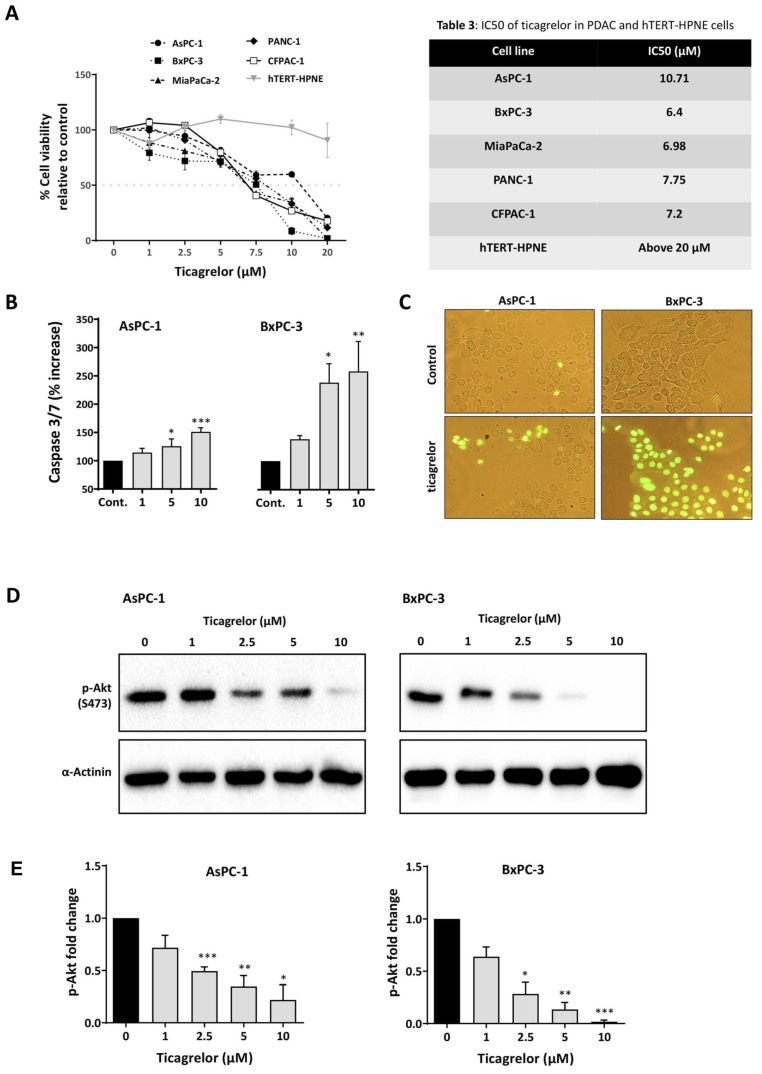
Ticagrelor treatment reduced PDAC cell viability and enhanced apoptosis through attenuating AKT activation in PDAC cells. (**A**) Relative cell viability in AsPC-1, BxPC-3, MiaPaCa-2, PANC-1, CFPAC-1 and h-TERT-HPNE cells upon treatment with ticagrelor (0–20 µM) for 72 h (*n* ≥ 3). Table 2 shows the IC_50_ (µM) of ticagrelor calculated using GraphPad PRISM 8 and log (inhibitor concentration) versus the normalised response (variable slope). (**B**) AsPC-1 and BxPC-3 cells were treated with ticagrelor (0–10 µM) for 12 h and analysed for apoptosis (caspase 3/7 activation). The columns represent the fold change in the level of activated caspase 3/7 relative to the control vehicle-treated cells, measured as described in the Methods and Materials section. Data are presented as mean ± SEM. One-way ANOVA with post-hoc Dunnett’s multiple comparison test was used to examine the significance of the mean; *n* ≥ 4, *** *p* < 0.001, ** *p* < 0.002, * *p* < 0.033. (**C**) Detection of caspase 3 activity in live cells treated with vehicle or ticagrelor 10 µM for 12 h. The caspase 3 substrate, once cleaved by caspase 3, formed a DNA dye, which stained the nucleus bright green. (**D**) Immunoblots showing the expression of p-AKT S473 in non-starved AsPC-1 and BxPC-3 cells treated with ticagrelor (0–10 µM) for 1 h. (**E**) The columns represent the fold change of p-AKT (S473) relative to the control vehicle-treated cells (*n* ≥ 3). Data are presented as mean ± SEM One-way ANOVA with post-hoc Dunnett’s multiple comparison test was used to examine the significance of the mean. *** *p* < 0.001, ** *p* < 0.002, * *p* < 0.033.

We next investigated the effect of ticagrelor on the proliferation of PDAC and normal pancreatic cells (Figure 4A, Table 2). Across PDAC cell lines, the IC_50_’s were less than 10 µM, while ticagrelor up to 20 µM did not show any cytotoxicity effect on the normal cells, hTERT-HPNE. To further validate the cancer selectivity of ticagrelor, AsPC-1 cells were grown in the same culture media used for hTERT-HPNE but without puromycin. Both cell lines were then treated with ticagrelor 10 µM; however, only AsPC-1 cells displayed sensitivity towards ticagrelor treatment (Figure S2C). This was a promising result as previous studies have established that ticagrelor plasma concentrations up to 10 µM are clinically tolerated [33]. PSB-0739, another P2Y12 inhibitor, also showed growth inhibitory effects (Figure S2D). As AKT is downstream of P2Y12 and is a known inhibitor of apoptosis [34], we investigated the effect of ticagrelor on apoptosis and AKT activity. Ticagrelor was found to induce a dose-dependent increase of caspase 3/7 (Figure 4B,C). Additionally, in non-serum-starved PDAC cells, ticagrelor caused a dose-dependent reduction in the expression level of phosphorylated AKT (Figure 4D,E).

### 3.5. Ticagrelor Synergised with Chemotherapy in PDAC Cells In Vitro

**Figure 5:**
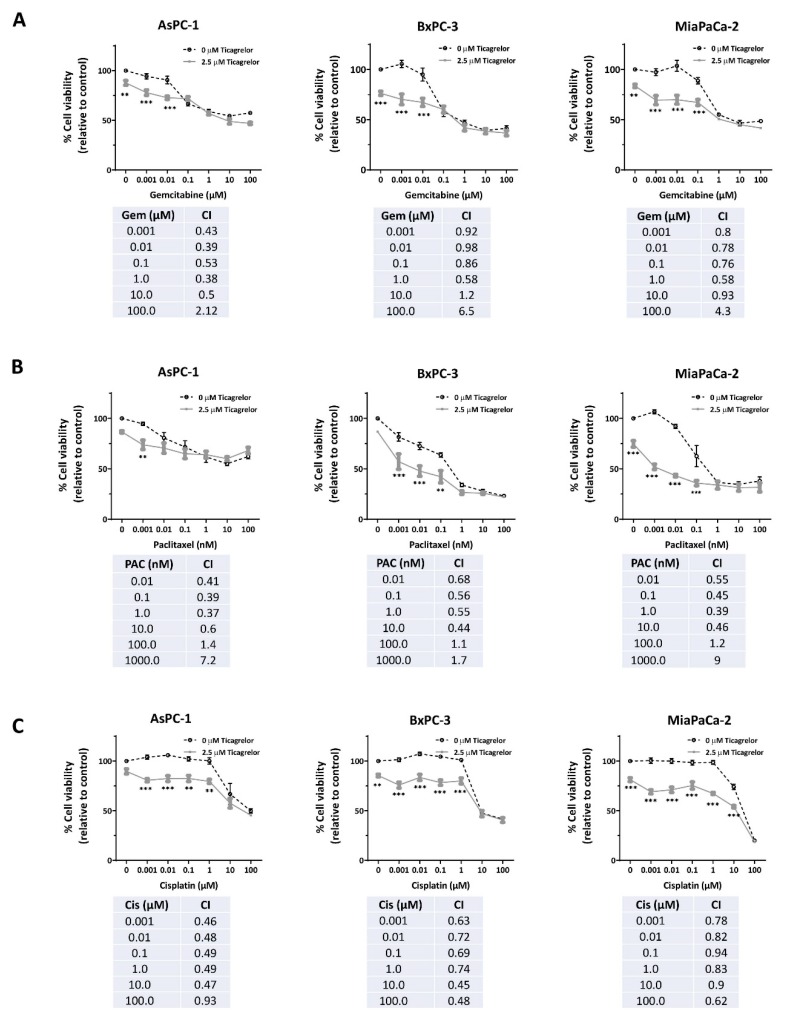
Ticagrelor synergised with chemotherapy in PDAC cells in vitro. (**A**–**C**) Relative cell viability in AsPC-1, BxPC-3 and MiaPaCa-2 upon treatment with gemcitabine (0–100 µM), paclitaxel (0–100 nM) and cisplatin (0–100 µM) as single agents or in combination with ticagrelor (2.5 µM) for 72 h. The significance of the difference between viability of cells treated with chemotherapy alone or in combination with ticagrelor was tested using two-way ANOVA with post-hoc Bonferroni’s multiple comparison test; *n* ≥ 3, *** *p* < 0.0001, ** *p* < 0.001. The tables below the graphs show the CI values calculated using Chou–Talalay’s method and can be interpreted as follow: CI < 0.9, synergism, CI > 1.1, antagonism, CI = 0.9–1.1, additive.

We next investigated, in vitro, the antitumour potential of ticagrelor in combination with PDAC chemotherapeutic drugs. Ticagrelor at a clinically relevant concentration (2.5 µM) [35,36] was combined with different concentrations of gemcitabine, paclitaxel and cisplatin. Dose-dependent growth inhibition was observed in AsPC-1, BxPC-3 and MiaPaCa-2 after 72 h of exposure to the chemotherapeutic agents (Figure 5A–C). AsPC-1 cells displayed the least sensitivity to the combined treatment (Figure 5A–C). The addition of ticagrelor improved the efficacies of the drugs in all tested PDAC cells. The combination therapies were largely synergistic, especially at low chemodrug concentrations (Figure 5A–C).

### 3.6. The Combination of Ticagrelor and Gemcitabine Significantly Reduced Tumour Growth In Vivo

**Figure 6:**
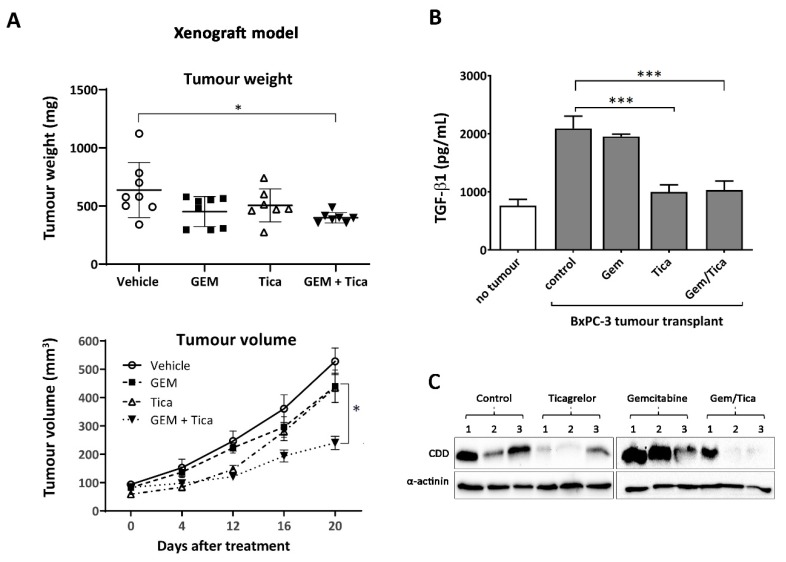
Combined treatment of ticagrelor (Tica) and gemcitabine (GEM) reduced tumour growth in vivo. (**A**) Effect of different treatments on tumour weight and volume in each NOD/SCID mouse bearing BxPC-3 tumours. The significance of the difference between tumour weight and volume in mice bearing BxPC-3 tumours treated with a vehicle control or gemcitabine, ticagrelor or gemcitabine plus ticagrelor was tested using ordinary one-way ANOVA (weight) or two-way ANOVA (volume) with post-hoc Dunnett’s multiple comparison test; *n* = 8 for vehicle, *n* = 8 for gemcitabine, *n* = 7 for ticagrelor, *n* = 7 for gemcitabine plus ticagrelor. The significance of the difference between the tumour volumes was calculated using the two-way ANOVA Dunnett’s multiple comparison test to examine the significance of the mean. *** *p* < 0.001, * *p* < 0.033. (**B**) The level of plasma TGF-β1 in NOD/SCID mice without tumours, or age-matched NOD/SCID mice with BxPC-3 tumours treated with vehicle, gemcitabine, ticagrelor or ticagrelor plus gemcitabine (*n* = 4). The significance of the difference between the TGF-β1 level was calculated using the two-way ANOVA Dunnett’s multiple comparison test to examine the significance of the mean. *** *p* < 0.001, * *p* < 0.033. (**C**) CDD expression in BxPC-3 tumour tissues extracted from mice treated with a vehicle control, ticagrelor, gemcitabine or ticagrelor plus gemcitabine (*n* = 3).

Gemcitabine alone or in combination with nab-paclitaxel is the standard of care in PDAC therapy [5]. Therefore, we tested the effect of gemcitabine in combination with ticagrelor on tumour growth, where in the xenograft model, BxPC-3 were transplanted into immune-deficient mice (NOD/SCID). After the tumour became palpable (3 weeks), mice were randomly distributed into four groups (vehicle control, gemcitabine, ticagrelor, gemcitabine plus ticagrelor). Ticagrelor has a shorter half-life in mice compared to humans, with a high concentration of ticagrelor (30–100 mg/kg) were required to achieve a maximum effect over 4 h post dosing [37]. Therefore, ticagrelor was administered twice a day every 12 h. As shown in Figure 6A and Figure S3A, only the combination therapy consistently and significantly reduced tumour growth. The mean tumour volume from the group was also significantly reduced compared to the gemcitabine treatment alone. Ticagrelor and gemcitabine as single agents had minimal effects on tumour growth.

Platelets are the main source of transforming growth factor-beta 1 (TGF-β1) in the circulation, which plays a crucial role in cancer metastasis [38,39]. Therefore, we examined the level of TGF-β1 in mice with no tumours and in mice with BxPC-3 xenografts treated with a vehicle, gemcitabine, ticagrelor or in combination. As shown in Figure 6B, ticagrelor alone or in combination with gemcitabine significantly reduced the plasma level of TGF-β1.

We previously showed that platelets promote CDD expression in pancreatic cancer cells [9]. CDD is known to deactivate gemcitabine and increase drug resistance. In treating pancreatic cancer patients, gemcitabine is given together with nab-paclitaxel as the latter has been shown to reduce CDD expression in tumour tissues [40,41]. In our study, as shown in Figure 6C, we found the addition of ticagrelor noticeably decreased the expression level of CDD in the tumour tissue from mice treated with ticagrelor or ticagrelor in combination with gemcitabine.

**Figure 7:**
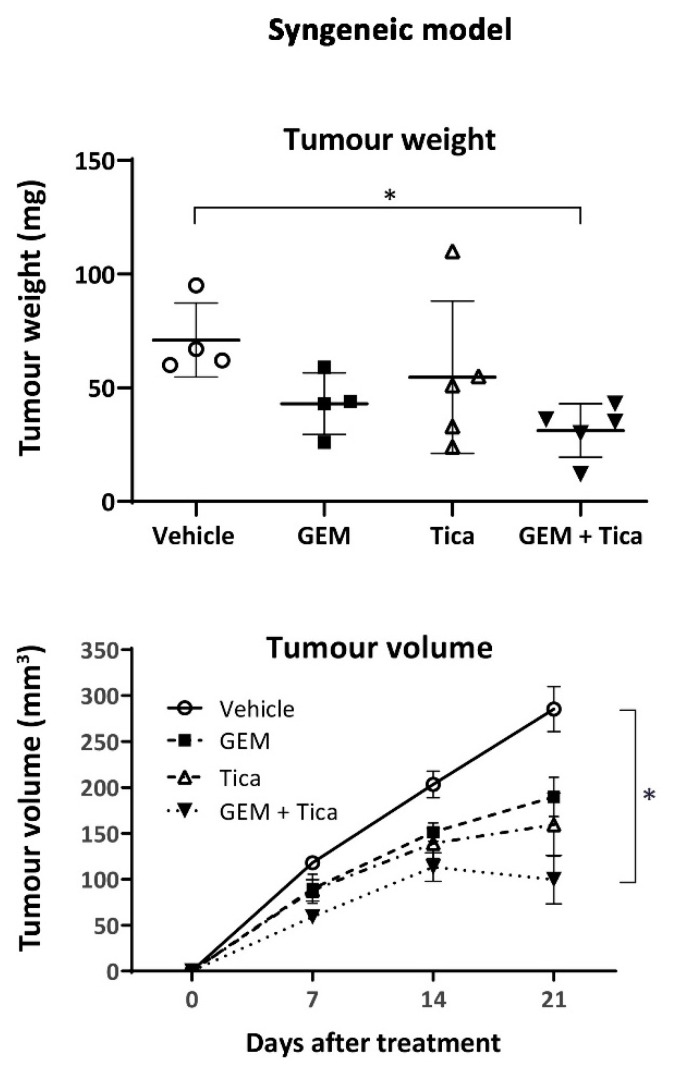
Ticagrelor potentiated gemcitabine activity in the syngeneic tumour mouse model. Effect of different treatments on tumour weight and volume in a syngeneic pancreatic cancer mouse model (C57BL6/J mice transplanted with MT4-2D). The significance of the difference between the tumour weight and volume in a syngeneic pancreatic cancer mouse model treated with vehicle control or gemcitabine, ticagrelor or gemcitabine plus ticagrelor was tested using ordinary one-way ANOVA (weight) or two-way ANOVA (volume) with post-hoc Dunnett’s multiple comparison test; *n* = 4 for vehicle, *n* = 4 for gemcitabine, *n* = 5 for ticagrelor, *n* = 5 for gemcitabine plus ticagrelor. The significance of the difference between the tumour volumes was calculated using two-way ANOVA Dunnett’s multiple comparison test to examine the significance of the mean. * *p* < 0.033.

Additionally, we examined the effect of the combined therapy on the growth of pancreatic cancer cells in immunocompetent mice. As shown in Figure 7 and Figure S3B, this treatment combination significantly reduced tumour growth, indicating that ticagrelor’s effect on tumour growth was not affected by a fully developed immune system. Importantly, the addition of ticagrelor to gemcitabine did not produce any noted adverse effects as the final weights and the haematological parameters were similar between the gemcitabine and combined therapy groups [42]. 

## 4. Discussion

Targeting ATP receptors in cancer cells or the ADP receptor P2Y12 in platelets have attracted overwhelming interest from cancer researchers recently [13,16]. However, the role of ADP and P2Y12 in cancer cells remains poorly investigated. Through functional and molecular studies, we demonstrated that the P2Y12 receptor was expressed in PDAC cells and was required for cancer cell proliferation. Targeting the P2Y12 receptor with ticagrelor repressed cancer cell growth and its presence synergised with several chemotherapeutic agents in vitro. The combination of ticagrelor and gemcitabine significantly reduced tumour growth in both the xenograft and syngeneic tumour mouse models tested.

P2Y12 belongs to a family of purinergic (P2) G-protein coupled receptors GPCRs [30]. We confirmed the expression of the P2Y12 protein in a group of pancreatic cancer cell lines. Using the anti P2Y12 monoclonal antibody EPR18611 (Abcam) against a specific peptide sequence of P2Y12 that does not share significant homology with P2Y1 or P2Y13, we were able to detect P2Y12 in cancer cells at the same molecular weight as in platelets (Figure 1A). The antibody performance was validated using a knockdown strategy where four different sequences of siRNA were used to suppress the expression of P2Y12 compared to siNeg-treated cancer cells (Figure S2A). The expression of P2Y12 was also confirmed using two different anti-P2Y12 antibodies (Figure S1). The gene expression was also validated using qPCR (Figure S5).

Several P2 receptor subtypes are involved in the transactivation of EGFR. For example, the ADP receptor P2Y1 and the ATP receptor P2Y2 have been shown to mediate oncogenic signalling through EGFR transactivation [19,20]. Here, our data clearly showed that extracellular ADP induced EGFR activation in PDAC cells. P2Y12 contributed to EGFR activation, potentially via several mechanisms. First, P2Y12 may induce EGFR transactivation through Src and matrix metalloproteases (MMP) axis as demonstrated with P2Y1 and P2Y2 [18,20]. Second, P2Y12 could regulate EGFR activation by promoting EGFR association with another member of the EGFR family, the human epidermal growth factor receptor 3 (HER3). Thus, inhibition of the purinergic receptor could reduce HER3 activation, and subsequently EGFR activation [19,43]. Third, P2Y12 could induce EGFR activation via Src, then the transactivated EGFR forms a multireceptor complex with P2Y12, leading to an increase in the downstream oncogenic signaling [44]. The specific mechanism of how P2Y12 contributes to EGFR activation remains to be determined in future studies.

ADP is known to elicit platelet activation through its interaction with P2Y1 and P2Y12 receptors. However, only the binding of P2Y12 leads to AKT activation, which is essential in platelet activation [30]. Our results demonstrated a similarity between P2Y12 signalling in platelets and PDAC cells since only the inhibition of P2Y12, but not P2Y1, reduced ADP-mediated AKT phosphorylation. Previous studies have indicated that P2 signalling promotes cancer invasion and chemotherapy resistance by supporting EMT in cancer cells [45]. Signalling through ATP, for example, can promote the upregulation of the mesenchymal transcriptional factor SNAIL1 in prostate cancer cells [46], whereas we previously demonstrated that ADP signalling promotes SLUG upregulation in PDAC cells [9]. SNAIL1 and SLUG (SNAIL2) belong to a family of mesenchymal transcriptional factors that can modulate EMT and chemotherapy resistance through controlling the expression of cell adhesion proteins (e.g., E-cadherin), other mesenchymal transcriptional factors (e.g., ZEB1) or drug metabolising enzymes and drug transporter proteins (e.g., CDD, ENT1) [5]. Here, our results showed for the first time that P2Y12 mediated the expression of the EMT-related factors SLUG and ZEB1. Interestingly, analysis of RNA sequencing expression data from tumours and normal samples from The Cancer Genome Atlas (TCGA) and Genotype-Tissue Expression (GTEx) projects using the web-based tool GEPIA revealed a positive correlation between the expression of P2Y12 and ZEB1 (Pearson correlation coefficient = 0.76, *p* < 0.05) in PDAC patients (Figure S4B,C). As recent data indicate that ZEB1 is a critical player in pancreatic cancer metastasis [47], substantially more investigations are needed to elaborate on the role of the P2Y12–ZEB1 axis in cancer invasion and metastasis.

Ticagrelor can attenuate AKT activation in platelets in response to a variety of platelet stimulants [48]. Here, our data suggested a novel effect for ticagrelor in PDAC cells through the reduction of the activity of EGF downstream effectors AKT and ERK. Inhibition of the P2Y1 also reduced EGF-induced AKT and ERK activation (Figure S1E), indicating that both P2Y1 and P2Y12 contributed to EGFR signalling. The inconsistent inhibitory effect of ticagrelor, unlike the results of P2Y12 siRNA knockdown, on the expression level of phosphorylated EGFR (p-EGFR Y1068) could have been due to the off-target effects of ticagrelor on ENT1. Ticagrelor is known to increase extracellular adenosine through the inhibition of ENT1-mediated adenosine cellular uptake [49]. Extracellular adenosine can also induce EGFR activation [50], thus the effect of ticagrelor on EGFR activation may be disrupted by the presence of adenosine.

The effects of ticagrelor on PDAC cell proliferation and apoptosis observed here are in accordance with a recent study where it was shown that P2Y12 protected platelets from apoptosis via the AKT-dependent inactivation of apoptosis regulators Bak and Bax [34]. Furthermore, our results showing minimal impact of ticagrelor on normal pancreatic duct cells were comparable to the previous Food and Drug Administration (FDA) data indicating ticagrelor up to 20 µM had a negligible toxicity on hepatocytes in vitro [33]. Ticagrelor has been reported to have several off-target effects that may be related to tumour growth [33,49,51,52]. Therefore, it is possible that the anticancer activity of ticagrelor is the result of a multitarget effect. Importantly, our results in the mouse models suggest that ticagrelor plus gemcitabine exerted a significant antitumour effect. As antitumour immune responses can be restrained by tumour-educated platelets [53], the tumour-suppressive impact of ticagrelor in the syngeneic model may be partly ascribed to its inhibitory effect on platelet function.

Ticagrelor use is associated with an increased bleeding risk. However, its short-term activity and recent FDA approval of a reversal agent may make it a more favourable choice than other antiplatelet agents in the context of cancer-associated thrombosis [5]. It is important to mention that several clinical trials failed to show a survival benefit of using anticoagulants for the primary or secondary prevention of venous thrombosis in pancreatic cancer, despite a reduction in the incidence of thrombotic events at the expense of the bleeding risk [6,7,8,9]. Therefore, antiplatelet medication, such as ticagrelor, may provide an alternative strategy to mitigate cancer-associated thrombosis, as well as conferring a direct anti-tumour activity, as highlighted by our study.

## 5. Conclusions

The tumour micro-environment, of which platelets are integral, is involved in cancer metastasis and chemotherapy resistance. Extensive research has reported that extracellular ADP in the tumour micro-environment can stimulate platelets through interaction with the platelet receptor P2Y12. In turn, activated platelets release biological factors supporting cancer progression. Our study revealed that pancreatic cancer cells also expressed a functional P2Y12, which is required for cell proliferation by promoting EGFR-dependent and independent AKT-mediated survival signalling. Subsequently, we demonstrated that blocking P2Y12 with the clinically available antiplatelet drug, ticagrelor, reduced cancer cell proliferation, activated apoptosis, and synergised with several chemotherapeutic agents in vitro. The addition of ticagrelor to gemcitabine significantly reduced tumour growth in both wildtype and immune-compromised xenograft mouse models, indicating that ticagrelor may be a promising option in PDAC therapy, and potentially for other cancers with similar pathologies and underlying mechanisms.

## Figures and Tables

**Table 1:** Combination index (CI): synergism (CI < 1), additive effect (CI = 1) and antagonism (CI > 1).

A. AsPC-1		B. BxPC-3	
Erlotinib (µM)	CI	Erlotinib (µM)	CI
0.001	0.43	0.001	0.88
0.01	0.42	0.01	0.84
0.1	0.5	0.1	0.91
1	0.62	1	0.8
10	1.4	10	1.1

**Table 2:** IC_50_ of ticagrelor in PDAC and hTERT-HPNE cells.

Cell Line	IC_50_ (µM)
AsPC-1	10.71
BxPC-3	6.4
MiaPaCa-2	6.98
PANC-1	7.75
CFPAC-1	7.2
hTERT-HPNE	Above 20 µM

## Supplementary Materials

The following are available online at https://www.mdpi.com/2072-6694/12/1/250/s1, Figure S1: Verification of P2Y12 expression using two different anti-P2Y12 antibodies, Figure S2: Verification of P2Y12 knockdown and its effect on the downstream target SLUG using four different siP2Y12 sequences, Figure S3: Image of all extracted tumour masses from mice, Figure S4: Correlation of P2Y12 expression with EGFR, ZEB1 and SLUG, Figure S5: P2Y12 gene expression in pancreatic cancer cell lines, Figure S6: Western blot files with densitometry data.

## Abbreviations

ADP	Adenosine diphosphate
ATP	Adenosine triphosphate
CDD	Cytidine deaminase
CI	Combination index
Cis	Cisplatin
EGFR	Epidermal growth factor receptor
EMT	Epithelial-mesenchymal transition
ENT1	Equiliberative nucleoside transporter 1
Gem	Gemcitabine
GEPIA	Gene expression profiling interactive analysis
GPCRs	G-protein coupled receptors
MMP	Matrix metalloproteases
Pac	Paclitaxel
PDAC	Pancreatic ductal adenocarcinoma
Tica	Ticagrelor
