# Supplementary material for: Antiplatelet Drug Ticagrelor Enhances Chemotherapeutic Efficacy by Targeting the Novel P2Y12-AKT Pathway in Pancreatic Cancer Cells
Source: Cancers (Basel). 2020 Jan 20;12(1):250. doi: 10.3390/cancers12010250 (PMC7016832; doi:10.3390/cancers12010250)
Supplement: Supplementary file 1 [file cancers-12-00250-s001.pdf]

Supplementary Materials:

Antiplatelet Drug Ticagrelor Enhances Chemotherapeutic Efficacy by Targeting the Novel P2Y12-AKT Pathway in Pancreatic Cancer Cells

Omar Elaskalani, Alice Domenichini, Norbaini Binti Abdol Razak, Danielle E. Dye, Marco Falasca and Pat Metharom

Materials and Methods

RNA extraction and cDNA synthesis

RNA was prepared from the pancreatic cell line hTert-HPNE and pancreatic cancer cell lines AsPC-1, MIA PaCa-2, BxPC-3, PANC1 and CFPAC-1. For each cell line, cells were harvested on three different days, pelleted, and pellets stored at −80 °C until use. Pellets (*n* = 3 for each cell line) where thawed and RNA extracted using the PureLink RNA Mini Kit (Ambion, Life Technologies, Thermofisher, Waltham, MA) according to the manufacturer's instructions. Briefly, cell pellets were re-suspended in lysis buffer, vortexed, and homogenised by passing through an 18 gauge needle. The lysed cell mixture was then combined with 70% ethanol and transferred to a spin cartridge. The spin cartridge was centrifuged at 12,000 *g* for 30 seconds (s) at room temperature (RT) and the flow-through discarded. Wash buffer was added and the spin cartridge centrifuged at 12,000 *g* for 30 s at RT. A DNAseI solution was then added to the spin cartridge matrix and incubated at RT for 15 minutes (min). Wash buffer was added and the spin cartridge centrifuged at 12,000 *g* for 30 s at RT. The flowthrough was discarded and the wash step repeated. The spin cartridge was transferred to a fresh 1.5 mL collection tube and RNA eluted with 100 µL of RNase-free water via centrifugation at 12,000 *g* for 60 s at RT. RNA concentration and purity was determined using the Nanodrop™ 1000 spectrophotometer (ThermoFisher Scientific).

1 µg of RNA was used for cDNA synthesis using the Tetro cDNA Synthesis Kit (Bioline, Meridian Bioscience, Memphis, TN) using a mixture of random hexamer and Oligo (dT) primers in a 20 mL reaction. Thermocycling conditions were 10 min at 25 °C, 30 min at 42 °C, 5 min at 85 °C and hold at 4 °C. cDNA was diluted 1:2 with DEPC-treated water and stored at −20 °C for short term storage.

Quantitative Real-Time polymerase chain reaction (qRT-PCR)

All qRT-PCRs were conducted using the 2× SensiFASTTM Sybr No-ROX (Bioline) and the CFX Connect Real-Time PCR Detection System (Biorad, Hercules, CA). The reference gene *GAPDH* (Glyceraldehyde 3-phosphate dehydrogenase) was amplified using the PrimePCR SYBR green assay GAPDH primers. producing a 147 base pair (bp) product (unique assay ID qHsaCED0038674, Biorad). *P2Y12* was amplified using primers situated within exon 3 (Forward; 5′-CACTGCTCTACACTGTCCTGT-3′; Reverse 5′-AGTGGTCCTGTTCCCAGTTTG-3′), amplifying a 190 base pair (bp) fragment. This region of the gene sequence is common to both *P2Y12* gene variants (variant 1—NM_022788.4, variant 2—NM_176876.3). qRT-PCR was performed using 7.5 µL 2× SensiFASTTM Sybr No-ROX, 1 µL 1:2 diluted cDNA, and 10 uM of each primer. Cycle conditions were: initial denaturation at 95 °C for 3 min followed by 40 cycles of 95 °C for 10s, 60 °C for 15s (and 72 °C for 20s. Melt curve analysis was performed at 95°C for 15 s, followed by ramping from 55 °C to 95 °C at 0.5 °C increments over 5 min. Melt curve analysis confirmed the presence of a single melt peak (indicating amplification of a single product) and amplicons were also electrophoresed on a 2% agarose gel to confirm amplification of a band of the expected size. Data were analysed by the delta-delta−Ct (ΔΔCt) method. Ct values of *P2Y12* were normalised against the Ct value of *GAPDH* for each sample (*n* = 3 sample per cell line); and these values in the pancreatic cancer cell samples were

then normalized against the average ΔCt of P2Y12 for the hTert-HPNE cells, which were used as the non-cancerous control. Expression of P2Y12 in pancreatic cancer cell lines is expressed as fold change compared to the hTert-HPNE cells [1,2].

Supplementary Figures

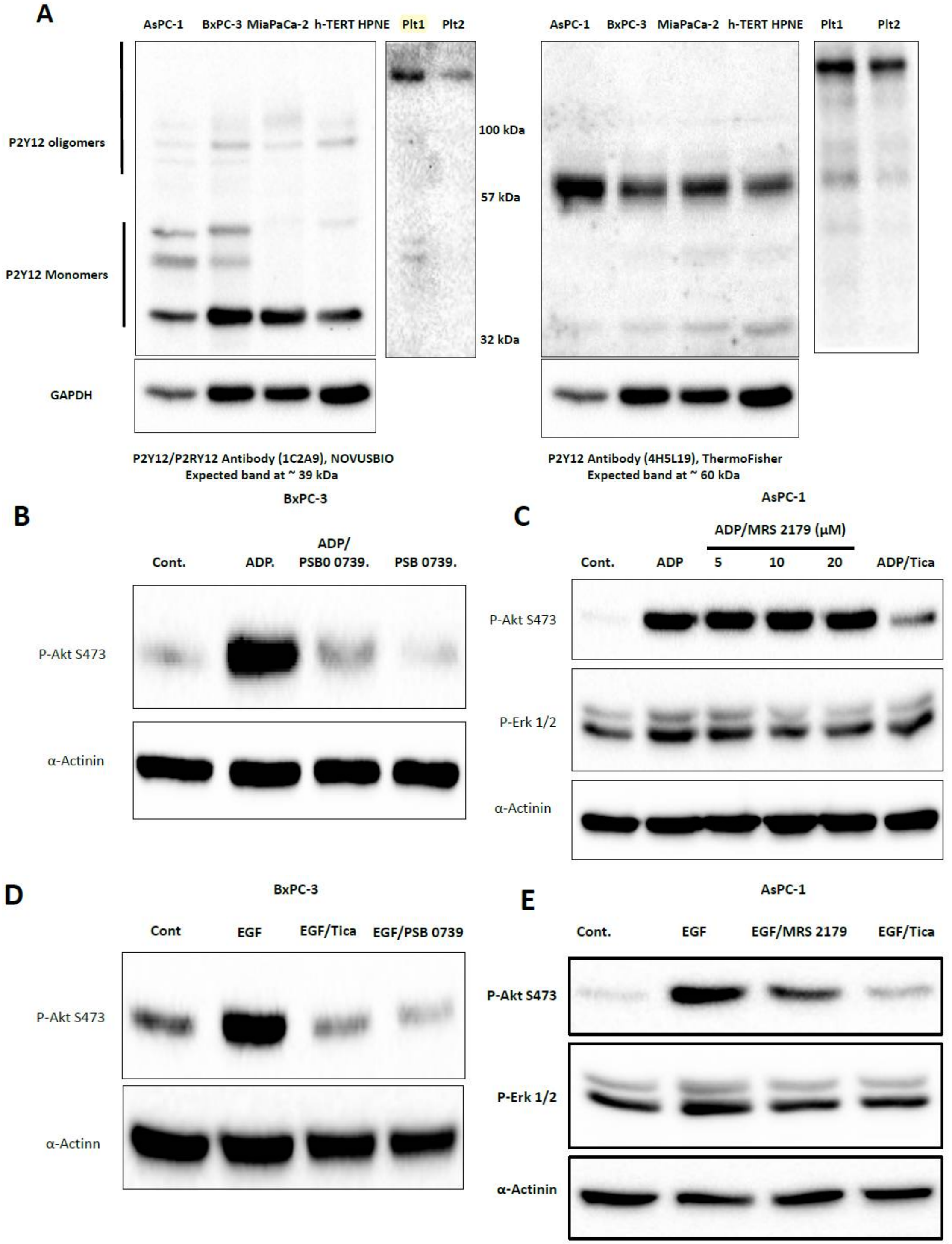

Figure S1. (**A**) Verification of P2Y12 expression using two different anti-P2Y12 antibodies. Platelets were used as a positive control (two different amounts of platelet lysates (20 and 10 μg of protein lysate per lane). As discussed in the methods and materials, platelet P2Y12 was detected mostly as

oligomers (> 100 kDa), while in PDAC cells P2Y12 was mostly detected as monomers (39–60 kDa). (**B**) ADP-stimulated AKT activation was reduced by another P2Y12 inhibitor; PSB 0739. Immunoblot shows the expression of p-AKT S473 in BxPC-3 cells treated with ADP (100 µM) combined with PSB 0739 (20 µM) for 30 min. (**C**) Inhibition of P2Y12, but not P2Y1, attenuated ADPstimulated AKT activation. Immunoblot shows the expression of p-AKT S473 and p-ERK 1/2 in AsPC-1 cells treated with ADP (100 µM) combined with the P2Y1 inhibitor, MRS 2179 (0-20 µM) or icagrelor (5 µM) for 30 min. (**D**) Inhibition of P2Y12 with PSB 0739 reduces EGF-stimulated AKT ctivation. Immunoblots show the expression of p-AKT S473 in BxPC-3 cells treated with EGF (10 ng/mL) combined with PSB 0739 (20 µM) or ticagrelor (5 µM) for 30 min. (**E**) Inhibition of P2Y1 with MRS 2179 reduces EGF-stimulated AKT and ERK activation. Immunoblot shows the expression of p-AKT S473 and p-ERK 1/2 in AsPC-1 cells treated with EGF (10 ng/mL) combined with MRS 2179 (20 µM) or ticagrelor (5 µM) for 30 min. Immunoblots (A-E) are representitve samples of 3 independentexperiments with similar results. Plt: platelet.

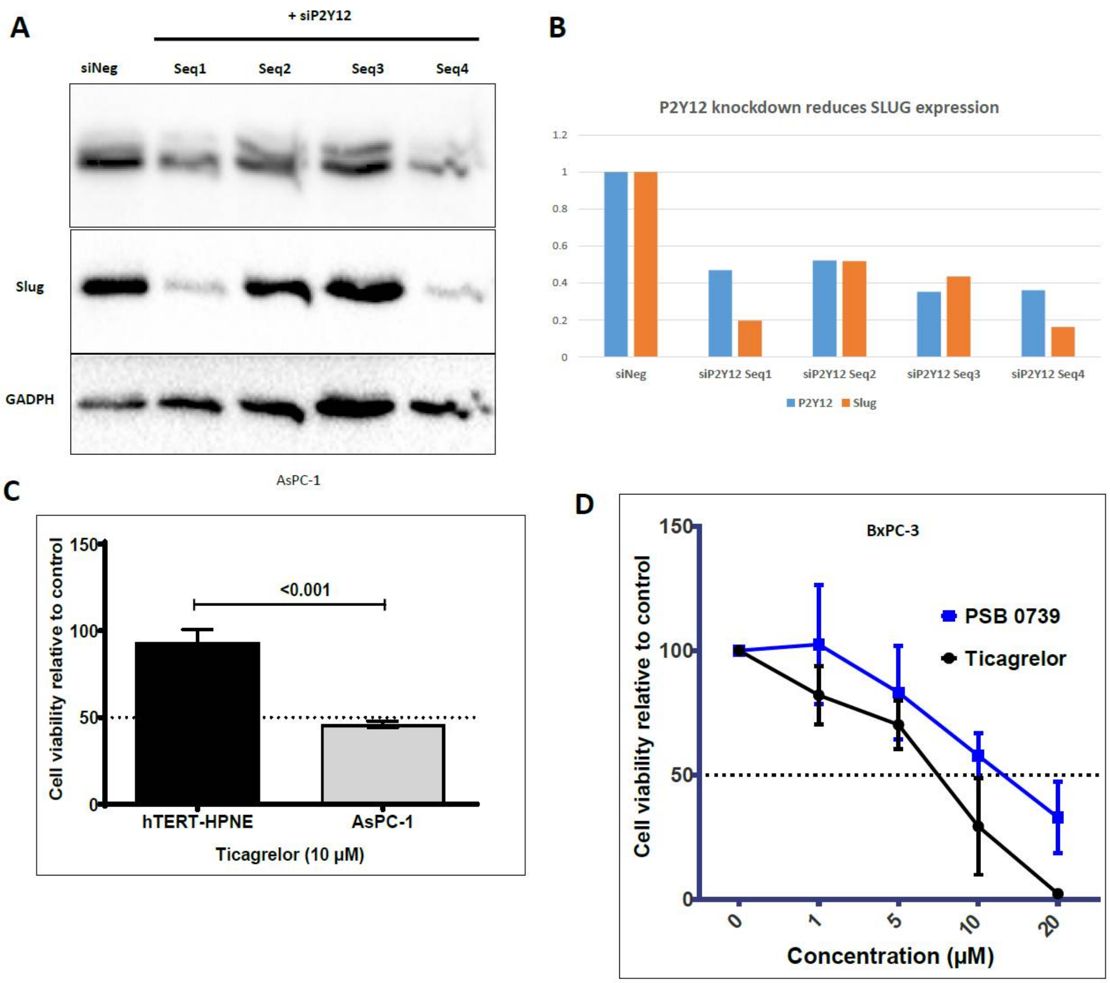

Figure S2. (**A**) Verification of P2Y12 knockdown and its effect on the downstream target SLUG using four different siP2Y12 sequences. FlexiTube GeneSolution (four different P2Y12 siRNAs) was obtained from Qiagen Pty Ltd, Australia, and the knockdown in AsPC-1 was progressed as described in the materials and methods section. (**B**) Fold change in P2Y12 and SLUG protein expression in AsPC-1 treated with four different P2Y12 siRNAs compred to siNeg transfected cells. (**C**) Ticagrelor has minimal cytotoxicity on normal pancreatic cells. AsPC-1 and h-TERT-HPNE were seeded in a 96- well plate in the same culture media, except for the selection antibiotic puromycin which is required for h-TERT-HPNE growth. After 24 h, cells were treated with ticagrelor (10 µM) for 72 h. Cell viability was measured as discussed in the methods and materials. Data are presented as mean ± SEM. The unpaired t-test was used to examine the significance of the mean. *N* = 5, *** $p < 0.001$. (**D**) PSB 0739

reduced cancer cell proliferation. Relative cell viability in BxPC-3 upon treatment with PSB 0739 or ticagrelor (0–20 μM) for 72 h (*n* = 3).

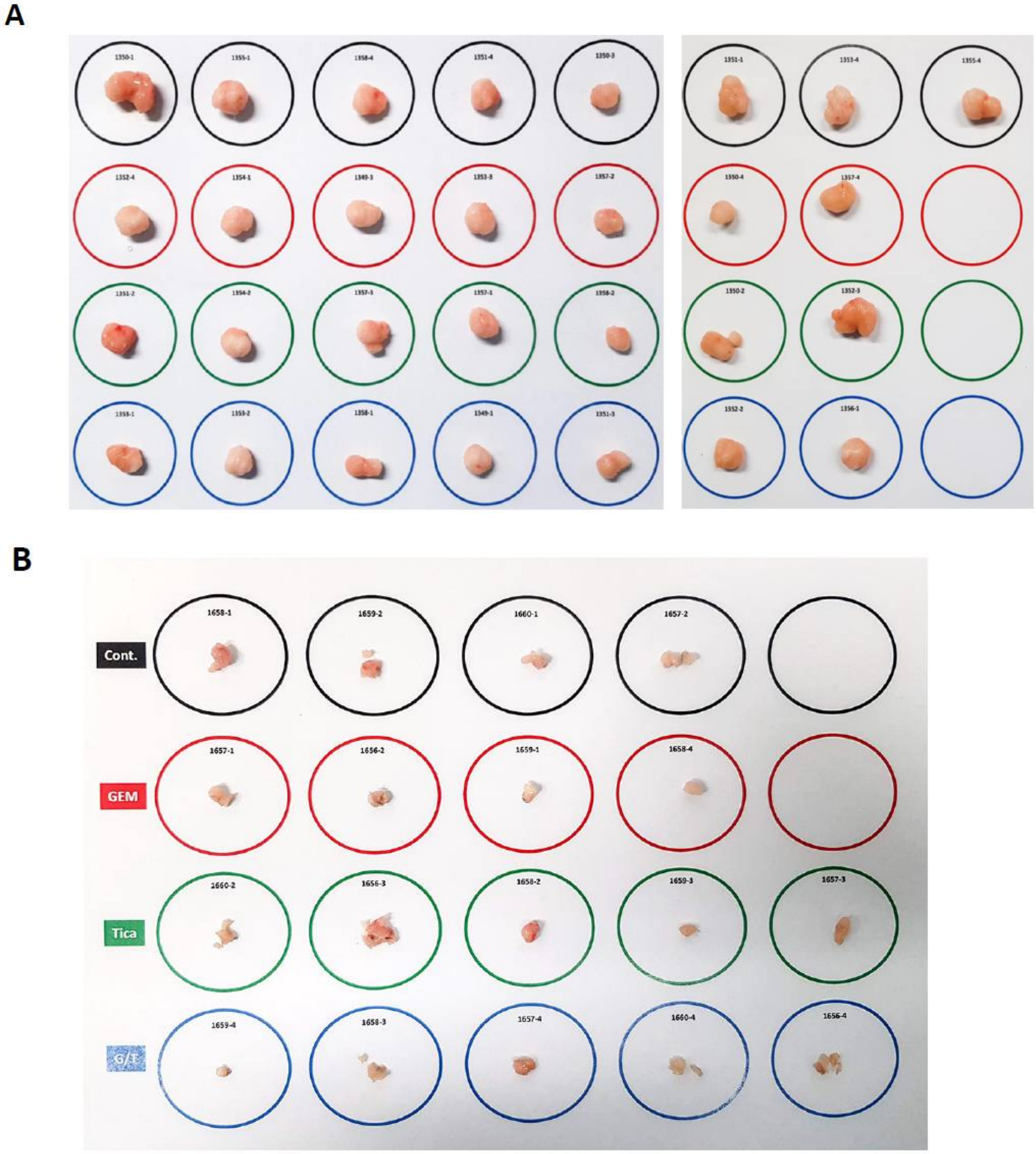

Figure S3. Image of all extracted tumour masses from NOD/SCID mice transplanted with human BxPC-3 cancer cells (**A**) and from wildtype mice transplanted with MT4-2D mouse cancer cells (**B**) treated with vehicle control, gemcitabine, ticagrelor or gemcitabine plus Ticagrelor as indicated in materials and methods.

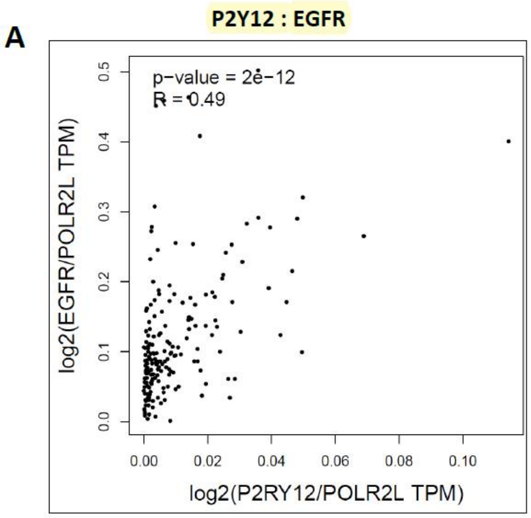

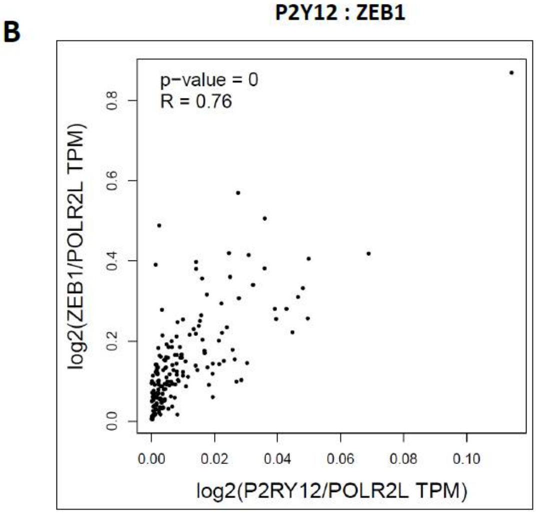

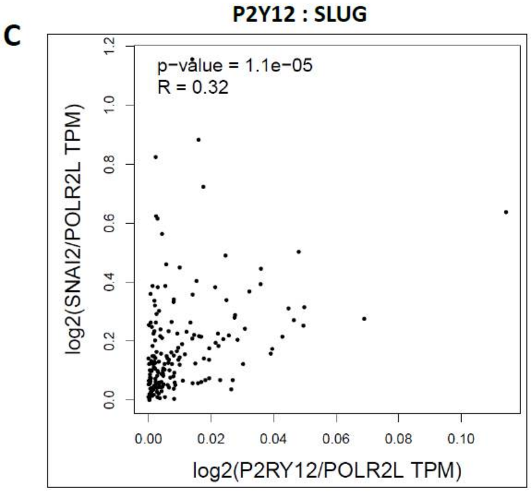

Figure S4. (A–C) Correlation of P2Y12 with EGFR (**A**), ZEB1 (**B**) and SLUG (**C**) in PDAC tissues from TCGA data. were calculated using the Pearson correlation coefficient. P2Y12 was normalised by the housekeeping gene POLR2L. *For A–C, data were analysed using the web-based tool GEPIA.

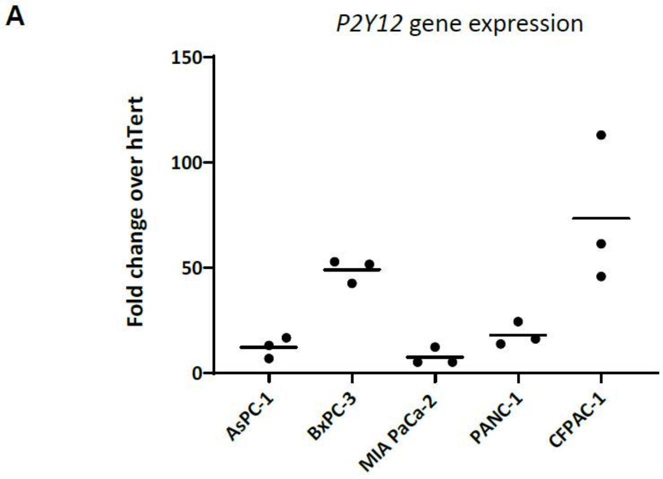

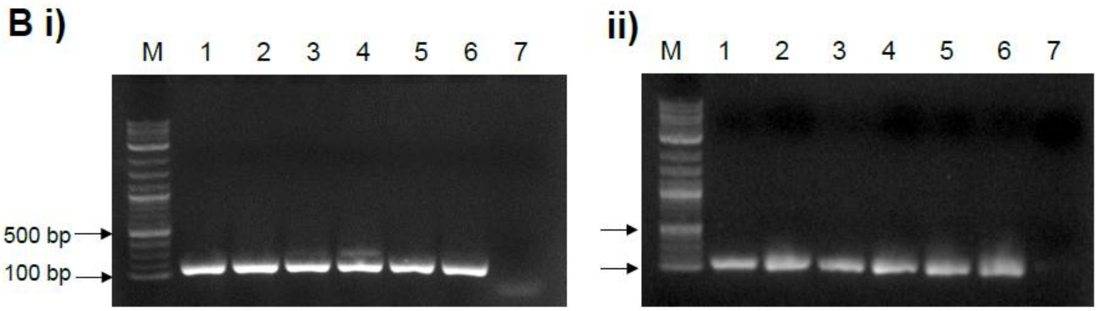

Figure S5. *P2Y12* gene expression in pancreatic cancer cell lines. **A**) qPCR was used to assess the expression of *P2Y12* in pancreatic cancer cell lines. Expression levels of *P2Y12* were first normalised to the Ct value of the reference gene GAPDH for each sample. Fold change was determined, with *P2Y12* expression in pancreatic cancer cell lines normalised to hTERT-HPNE expression (fold change = 1). Mean ± SE of 3 biological replicates are shown. Given the small number of replicates, statistical analyses was not performed. **B**) Specificity of the qPCR was assessed by analysing one biological replicate via agarose gel electrophoresis (2%). *P2Y12* amplicons were approximately 190 bp (i) and *GAPDH* amplicons 147 bp (ii); with a single band present in all samples. 1—hTert-HPNE, 2—AsPC-1, 3—BxPC-3, 4—MIA PaCa-2, 5—PANC1, 6—CFPAC-1, 7—control (no template).

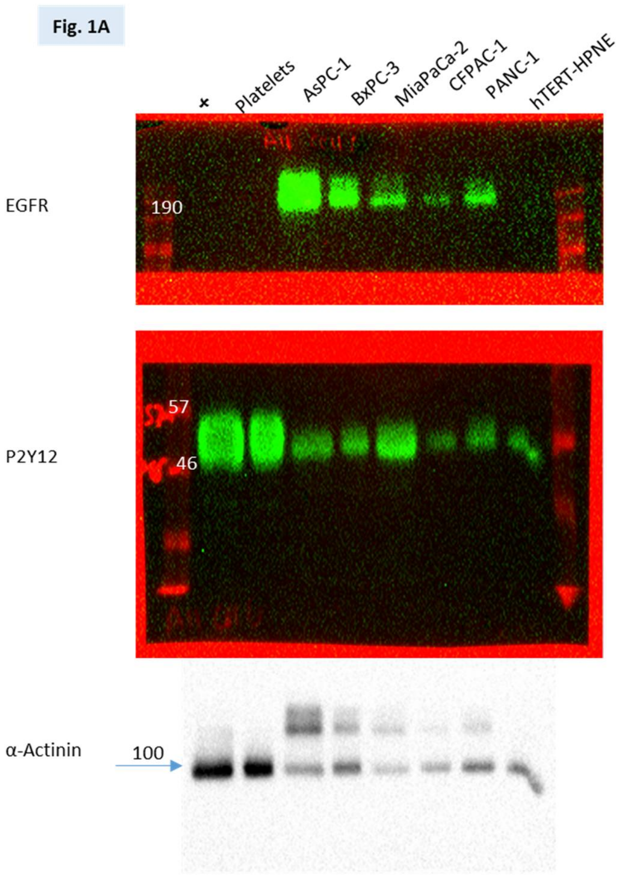

*Band intensity relative to house keeping protein is provided in Fig.1 (B-C)

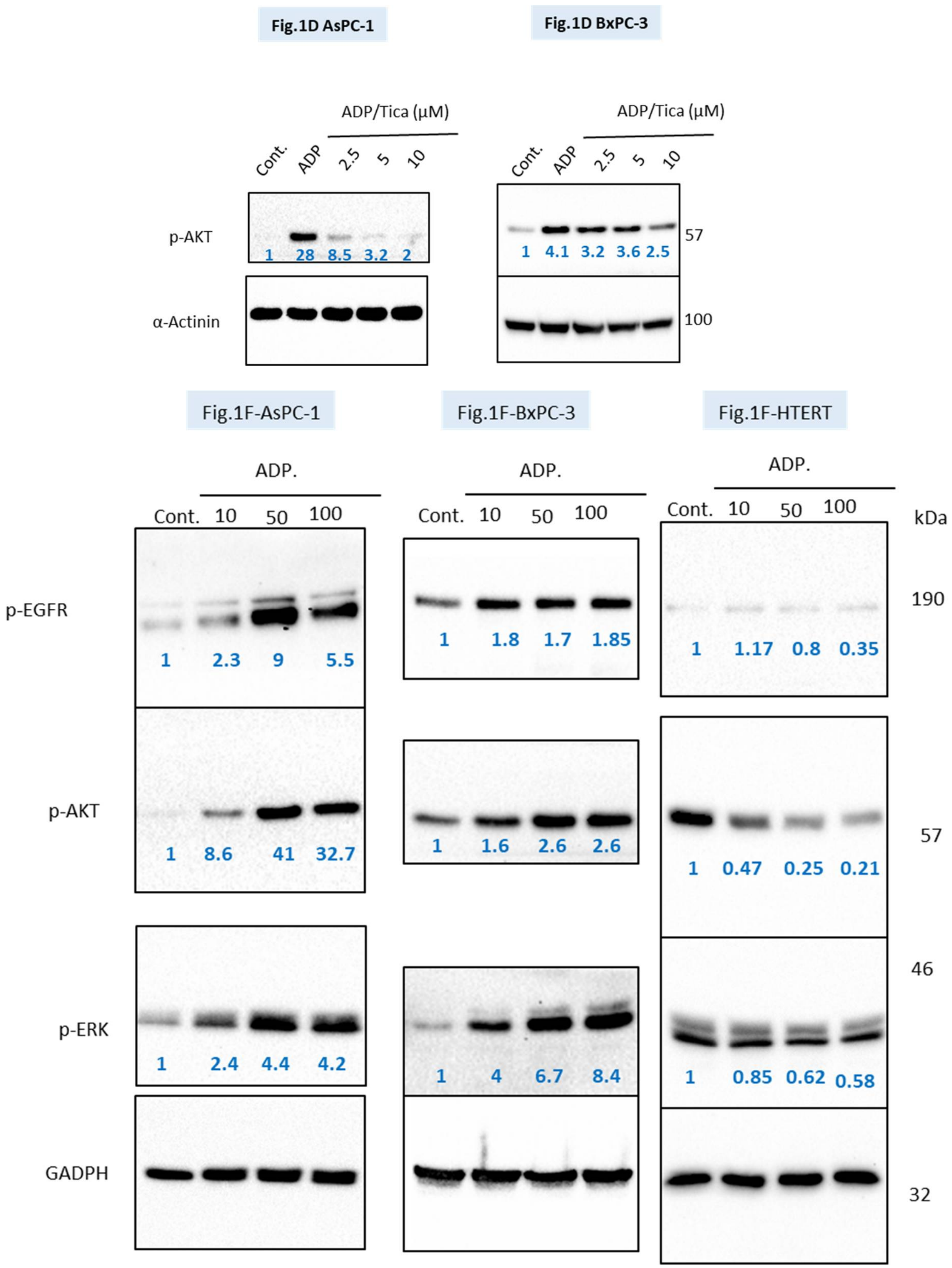
Fig.1D AsPC-1
Fig.1D BxPC-3
ADP/Tica (μM)
Cont.
ADP
2.5
5
10
p-AKT
1 28 8.5 3.2 2
1 4.1 3.2 3.6 2.5
57
α-Actinin
100
Fig.1F-AsPC-1
Fig.1F-BxPC-3
Fig.1F-HTERT
ADP.
Cont. 10 50 100
kDa
p-EGFR
1 2.3 9 5.5
1 1.8 1.7 1.85
1 1.17 0.8 0.35
190
p-AKT
1 8.6 41 32.7
1 1.6 2.6 2.6
1 0.47 0.25 0.21
57
46
p-ERK
1 2.4 4.4 4.2
1 4 6.7 8.4
1 0.85 0.62 0.58
GADPH
32

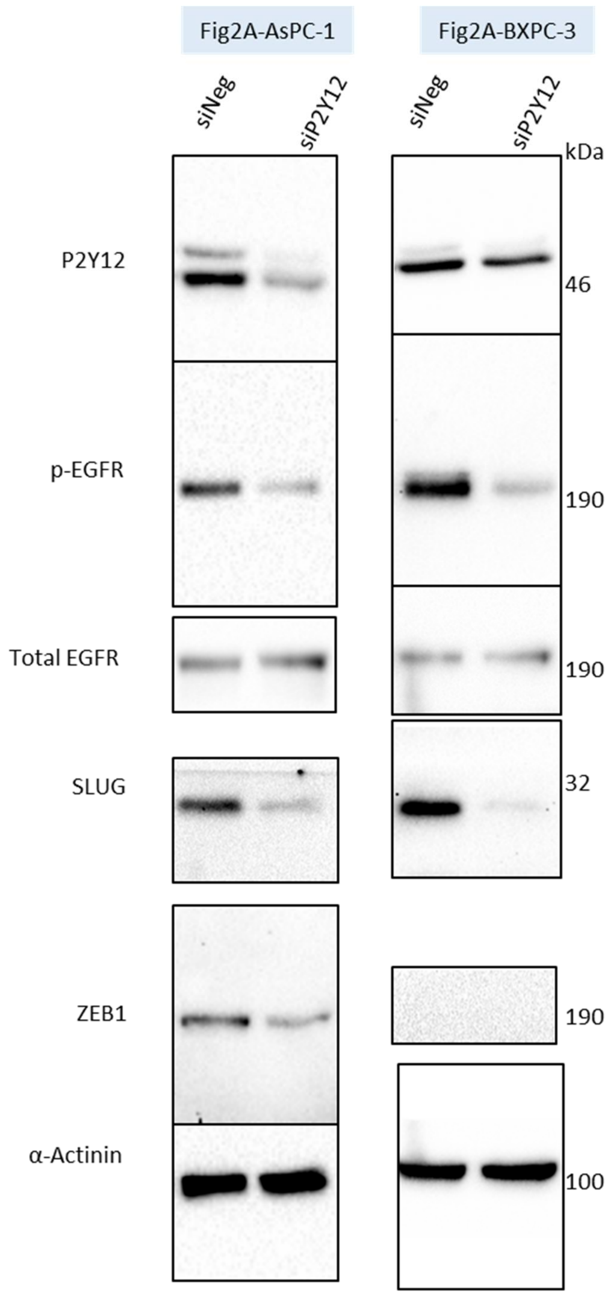

*Band intensity relative to house keeping protein is provided in Fig.2 (B-C)

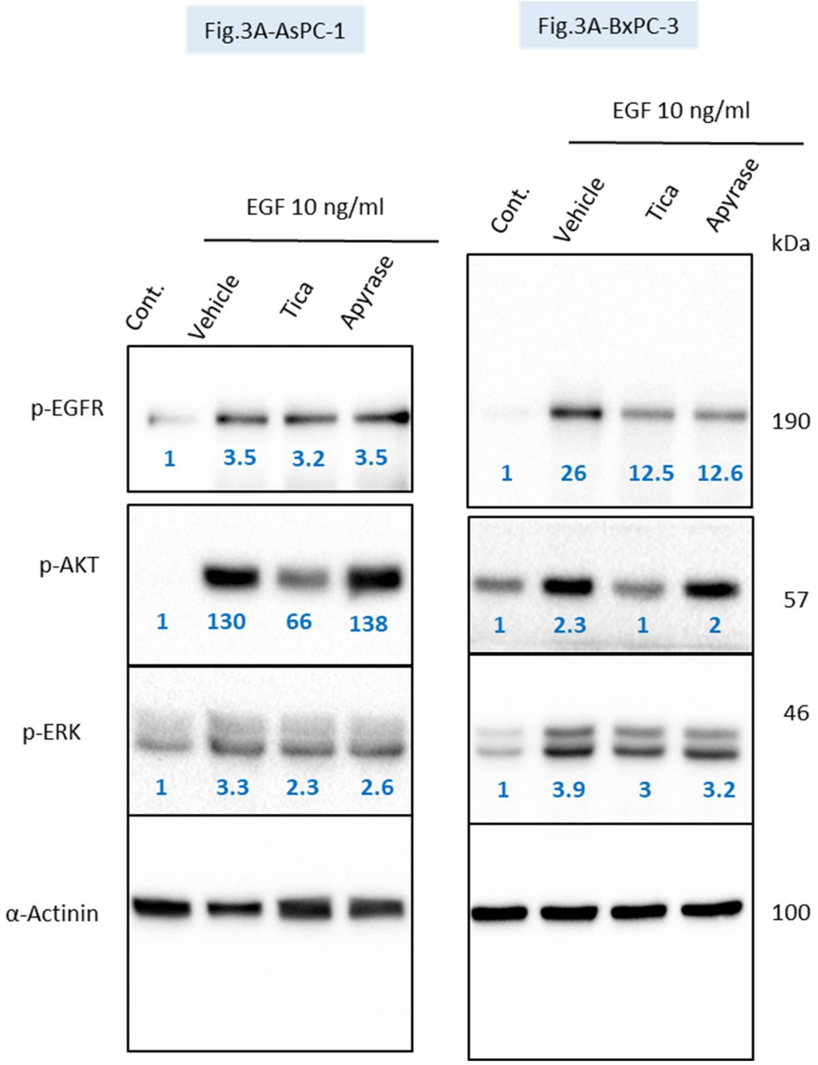
Fig.3A-AsPC-1
Fig.3A-BxPC-3
EGF 10 ng/ml
Cont.
Vehicle
Tica
Apyrase
kDa
p-EGFR
1 3.5 3.2 3.5
1 26 12.5 12.6
190
p-AKT
1 130 66 138
1 2.3 1 2
57
46
p-ERK
1 3.3 2.3 2.6
1 3.9 3 3.2
α-Actinin
100

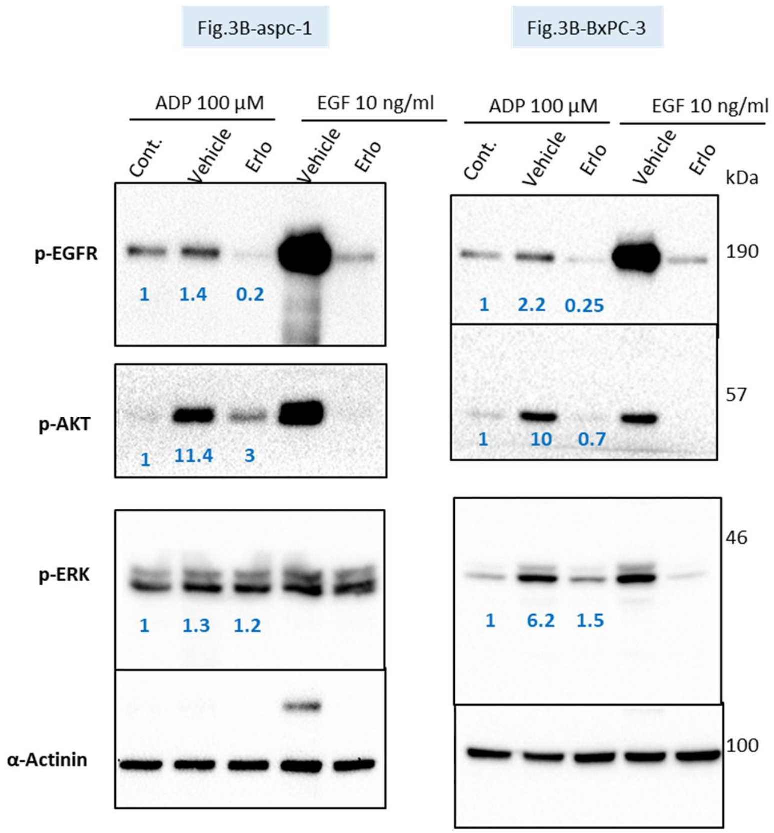
Fig.3B-aspc-1
Fig.3B-BxPC-3
ADP 100 μM
EGF 10 ng/ml
Cont.
Vehicle
Erlo
Vehicle
Erlo
kDa
p-EGFR
1 1.4 0.2
1 2.2 0.25
190
p-AKT
1 11.4 3
1 10 0.7
57
p-ERK
1 1.3 1.2
1 6.2 1.5
46
α-Actinin
100

Fig.3D-aspc-1

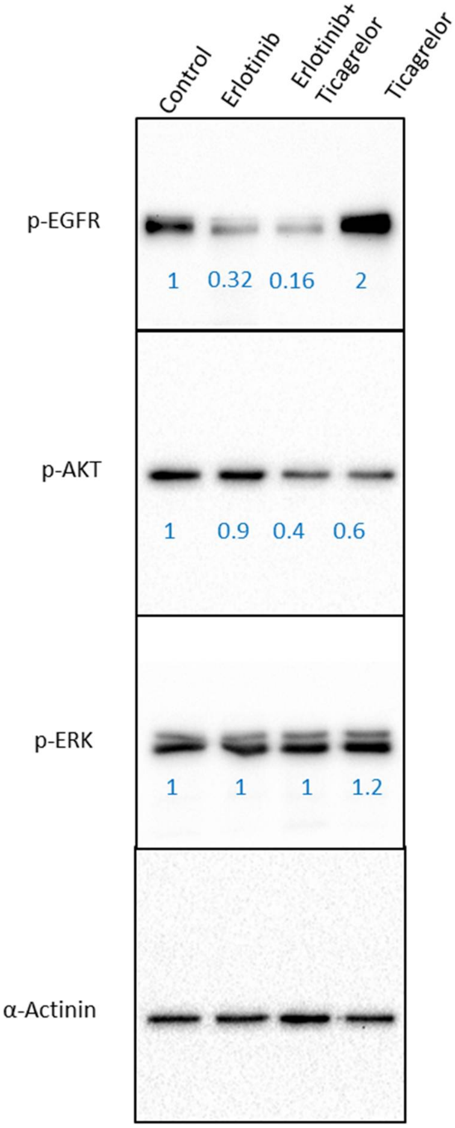

Fig.3D-BxPC-3

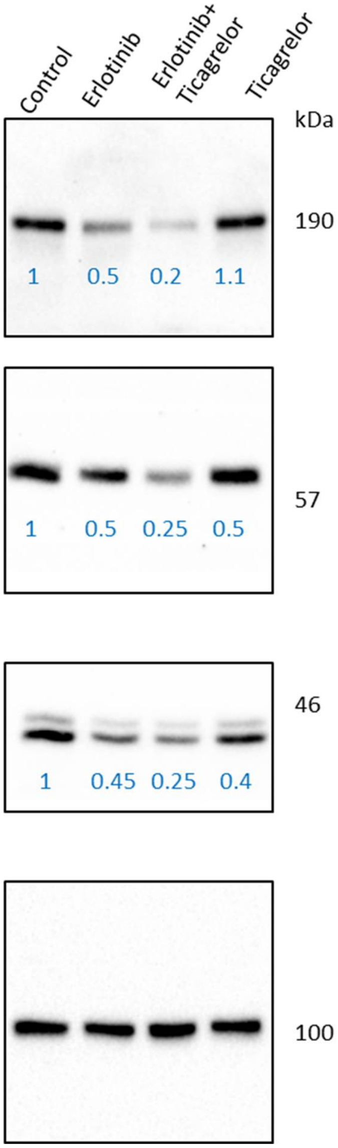

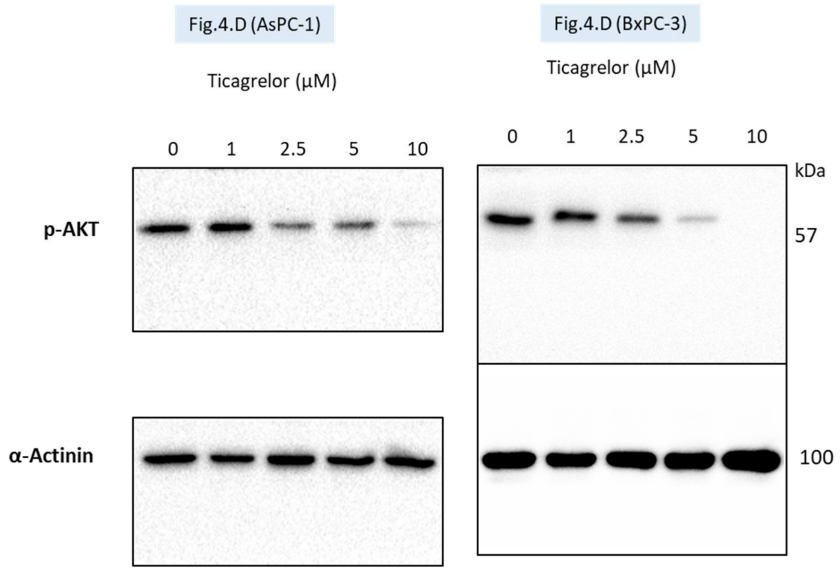

*Band intensity relative to house keeping protein is provided in Fig.4 D

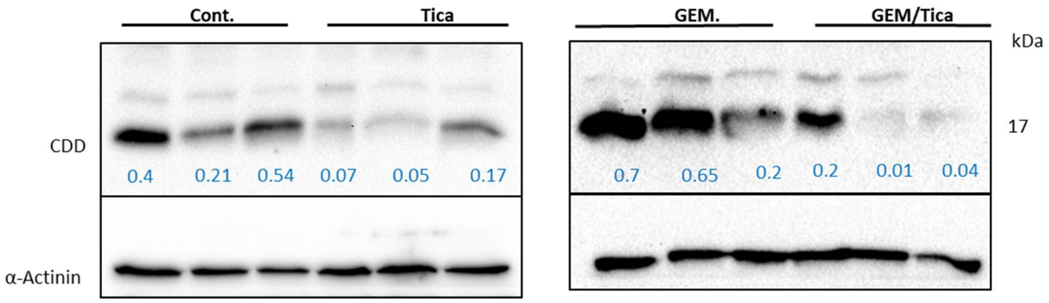

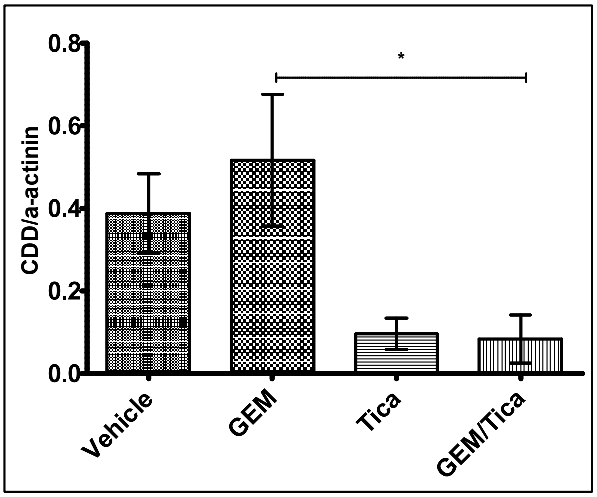

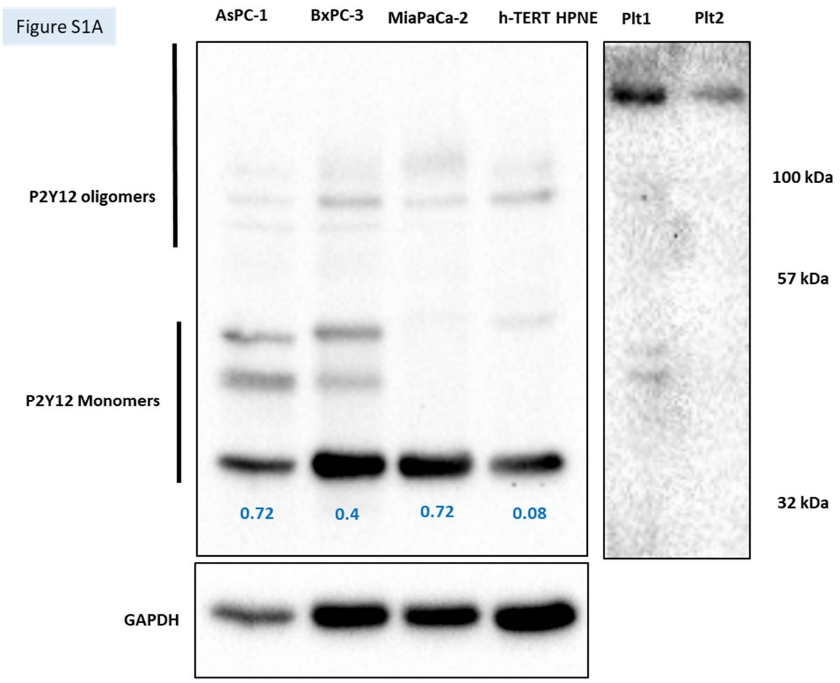

P2Y12/P2RY12 Antibody (1C2A9), NOVUSBIO
Expected band at ~ 39 kDa

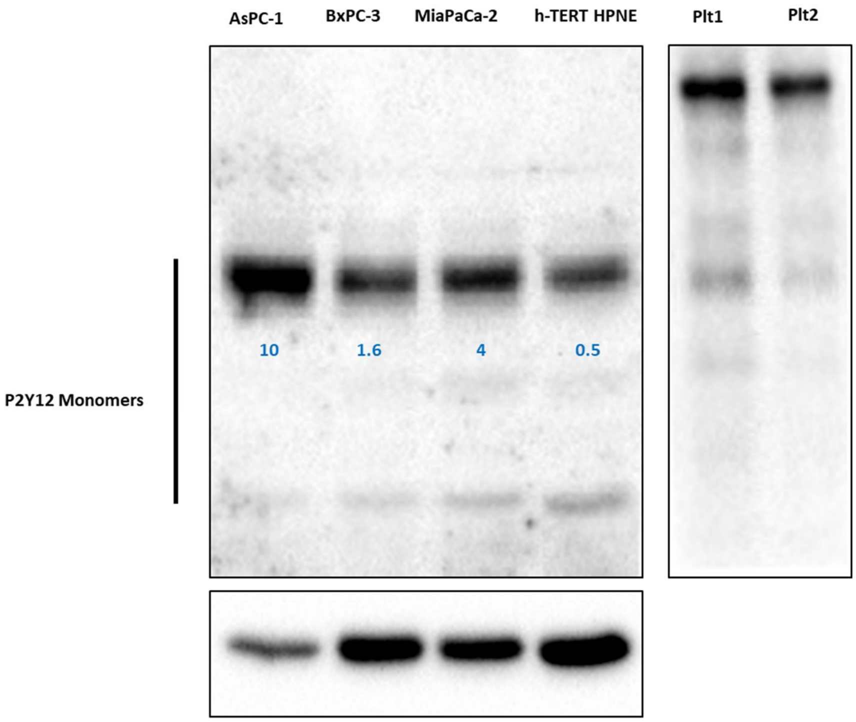

P2Y12 Antibody (4H5L19), ThermoFisher
Expected band at ~ 60 kDa

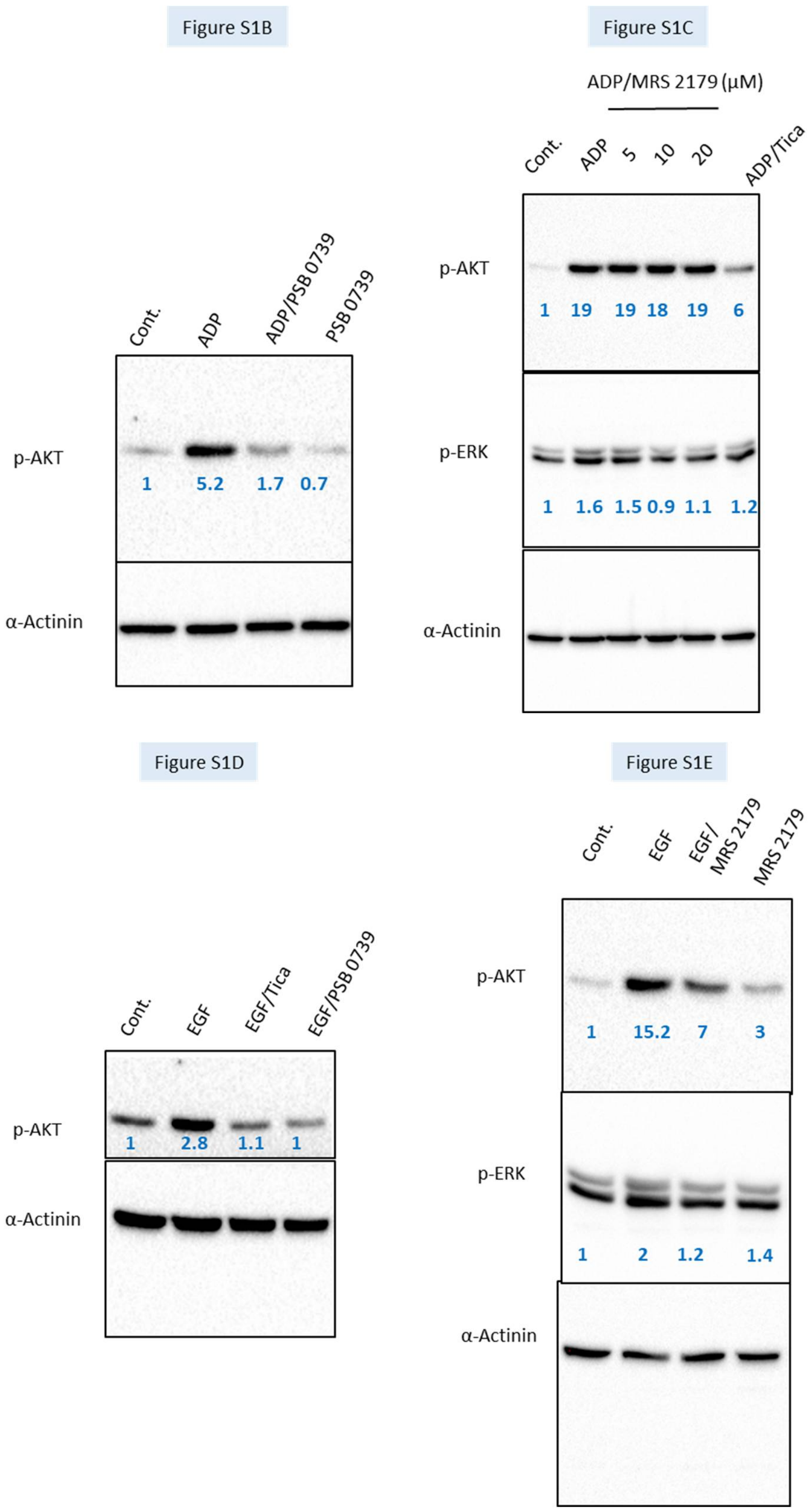

Figure S6. Western blot files with densitometry data.

Reference

1 Ding, Z.; Bynagari, Y.S.; Mada, S.R.; Jakubowski, J.A.; Kunapuli, S.P. Studies on the role of the extracellular cysteines and oligomeric structures of the P2Y12 receptor when interacting with antagonists. *J. Thromb. Haemost* **2009**,7, 232–234.

2 Savi, P.; Zachayus, J.-L.; Delesque-Touchard, N.; Labouret, C.; Hervé, C.; Uzabiaga, M.-F.; Pereillo, J.-M.; Culouscou, J.-M.; Bono, F.; Ferrara, P.; et al. The active metabolite of Clopidogrel disrupts P2Y12 receptor oligomers and partitions them out of lipid rafts. *Proc. Natl. Acad. Sci. USA* **2006**, 103, 11069–11074.

© 2020 by the authors. Licensee MDPI, Basel, Switzerland. This article is an open access article distributed under the terms and conditions of the Creative Commons Attribution (CC BY) license (http://creativecommons.org/licenses/by/4.0/).
